# Role of antiangiogenic agents in first-line treatment for advanced NSCLC in the era of immunotherapy

**DOI:** 10.1186/s12885-022-10446-1

**Published:** 2023-01-21

**Authors:** Lan-Lan Pang, Jia-Di Gan, Yi-Hua Huang, Jun Liao, Wei-Tao Zhuang, Wael-Abdullah-Sultan Ali, Shao-Dong Hong, Li Zhang, Wen-Feng Fang

**Affiliations:** Department of Medical Oncology, State Key Laboratory of Oncology in South China, Collaborative Innovation Center for Cancer Medicine, Sun Yat-Sen University Cancer Center, Guangzhou, 510060 People’s Republic of China

**Keywords:** Non-small cell lung cancer, Immunotherapy, Chemotherapy, Combination, Antiangiogenic agents, Meta-analysis

## Abstract

**Background & objective:**

“Anti-angiogenetic drugs plus chemotherapy” (anti-angio-chemo) and “immune checkpoint inhibitors plus chemotherapy” (ICI-chemo) are superior to traditional chemotherapy in the first-line treatment of patients with advanced non-small-cell lung cancer (NSCLC). However, in the absence of a direct comparison of ICI-chemo with anti-angio-chemo, the superior one between them has not been decided, and the benefit of adding anti-angiogenetic agents to ICI-chemo remains controversial. This study aimed to investigate the role of antiangiogenic agents for advanced NSCLC in the era of immunotherapy.

**Methods:**

Eligible randomized controlled trials (RCTs) comparing chemotherapy versus therapeutic regimens involving ICIs or anti-angiogenetic drugs were included. Outcomes included progression-free survival (PFS), overall survival (OS), objective response rate (ORR), and rate of grade 3–4 toxicity assessment. R-4.3.1 was utilized to perform the analysis.

**Results:**

A total of 54 studies with a sample size of 25,046 were finally enrolled. “Atezolizumab + Bevacizumab + Chemotherapy” significantly improved the ORR compared with “Atezolizumab + Chemotherapy” (Odds ratio (OR) = 2.73, 95% confidence interval (CI): 1.27–5.87). The trend also favored “Atezolizumab + Bevacizumab + Chemotherapy” in PFS and OS (hazard ratio (HR) = 0.71, 95% CI: 0.39–1.31; HR = 0.94, 95% CI: 0.77–1.16, respectively). In addition, “Pembrolizumab + Chemotherapy” and “Camrelizumab + Chemotherapy” significantly prolonged the PFS compared to “Bevacizumab + Chemotherapy” (HR = 0.65, 95% CI: 0.46–0.92; HR = 0.63, 95% CI: 0.41–0.97; respectively). Meanwhile, “Pembrolizumab + Chemotherapy” and “Sintilimab + Chemotherapy” yielded more OS benefits than “Bevacizumab + Chemotherapy” (HR = 0.69, 95% CI: 0.56–0.83; HR = 0.64, 95%CI: 0.46–0.91; respectively). Scheme between “Atezolizumab + Bevacizumab + Chemotherapy” and “Atezolizumab + Chemotherapy” made no significant difference (OR = 1.18, 95%CI: 0.56–2.42) concerning the rate of grade 3–4 toxicity. It seemed that ICI-chemo yielded more improvement in quality-adjusted life-year (QALY) than “Bevacizumab + Chemotherapy” in cost-effectiveness analysis.

**Conclusion:**

Our results suggest that ICI-chemo is associated with potentially longer survival, better cost-effectiveness outcomes, and comparable safety profiles than anti-angio-chemo. Also, adding bevacizumab to ICI-chemo seemed to provide additional therapeutic benefits without adding treatment burden. Our findings would supplement the current standard of care and help the design of future clinical trials for the first-line treatment of patients with advanced NSCLC.

**Supplementary Information:**

The online version contains supplementary material available at 10.1186/s12885-022-10446-1.

## Background

Lung cancer is the leading cause of cancer-related mortality worldwide [1]. Novel therapeutic approaches are urgently needed after the arrival of a “chemotherapy efficacy plateau” for advanced non-small cell lung cancer (NSCLC) [2]. Angiogenesis plays a critical role in tumor growth and metastasis. Key angiogenesis pathway is mainly inhibited via vascular endothelial growth factor (VEGF)/VEGF receptor signaling, either at the ligand level (e.g. bevacizumab) or at the receptor level (e.g. ramucirumab) or by the small-molecule small tyrosine kinase inhibitors [TKIs] (e.g. sorafenib) [3, 4]. Notably, bevacizumab plus platinum-based doublet chemotherapy has been approved for the treatment of advanced non-squamous NSCLC in the first-line setting [5].

The application of Ipilimumab in the first-line treatment of advanced NSCLC opened a new era of immunotherapy [6]. Chemotherapy elicits anti-tumor effects through the release of potentially immunogenic tumor antigens, which might result in additional immunotherapy activity and synergistic effect [7, 8]. The combination of Pembrolizumab and platinum-based doublet chemotherapy has been approved as a first-line treatment strategy for advanced non-squamous NSCLC patients without actionable genetic mutation in April 2019 [9]. Noteworthy, anti-programmed death 1 (PD-1) antibody and anti-cytotoxic T-lymphocyte antigen 4 (CTLA-4) antibody take distinct but complementary action. It was particularly critical in recruiting effective antitumor immunity and avoiding alternative exhausting pathway [10–12]. Consequently, anti-PD-1 plus CTLA-4 antibody is deemed to play a vital role in the era of immunotherapy. The efficacy of ICIs may be enhanced with the addition of anti-angiogenetic drugs via reversing VEGF-mediated immune-suppression [13, 14]. The landmark study— IMPOWER 150 trial, had firstly elucidated the superior efficacy of adding bevacizumab to ICI-chemo [15].

Anti-angiogenetic drugs plus chemotherapy (anti-angio-chemo) and ICI plus chemotherapy (ICI-chemo) are superior to chemotherapy alone in the first-line treatment for advanced NSCLC [3, 16]. However, the absence of head-to-head trials comparing ICI-chemo with anti-angio-chemo make no conclusion in which regimen is superior. Furthermore, the benefit of adding anti-angiogenetic agents to ICI-chemo remains controversial. In this study, we enrolled randomized-controlled trials (RCTs) and conducted a Bayesian Network Meta-analysis (NMA) to explore the above-mentioned matters. Besides, we reviewed the published articles concerning the cost-effectiveness analysis.

## Methods

This systematic review and NMA was performed in accordance with the PRISMA Extension Statement for Reporting Systematic Reviews Incorporating Network Meta-analyses guidelines of Health Care Interventions (Supplementary material 1). The protocol for this study was registered in the Prospective Register of Systematic Reviews (CRD42022309295) to ensure transparency.

### Data sources

Two authors (L.L.P and J.D.G) independently searched the records in the electronic database of PubMed, EMBASE, The Cochrane Library, and Web of science. The searching terminal date was June 2nd, 2021. Searching terms focused on “NSCLC”, “antiangiogenic agents”, and “ICIs” with the restriction of clinical trial. If necessary, an additional manual search of related literature in the reference list would be carried out to enroll any relevant publications. The datasets utilized in this analysis could be obtained from the corresponding author upon request. Records were imported into EndnoteX9 software to eliminate duplications. The detailed strategy was presented in Supplementary material 2.

### Trial selection criteria and trial identification

Two authors (L.L.P and Y.H.H) independently reviewed the titles, abstracts, and keywords of the identified citations to select appropriate articles for full review. Any disagreement was resolved by consensus. Trials would be eligible only if meeting all the following criteria: 1) treatment-naïve patients with stage IIIB/IV NSCLC; 2) eligible RCTs comparing ICI-chemo or anti-angio-ICI versus the platinum chemotherapy alone; or involving the addition of antiangiogenic drugs into ICI-chemo; 3) full-text publications or conference abstract. Publications would be disregarded if meeting any of the following criteria: 1) any single perioperative chemotherapy, neoadjuvant or adjuvant chemotherapy, or radio-chemotherapy; 2) no first-line treatment; 3) non-accessible outcome.

### Outcomes and data extraction

Two authors (L.L.P and J.L) independently performed data extraction and any discrepancies were eliminated by consensus. Data for the eligible trials related to basic characteristics were extracted.

The primary outcome included progression disease survival (PFS), overall survival (OS), objective response rate (ORR), and rate of grade3-4 toxicity. PFS was defined as the time interval from randomization to disease progression or death, whichever occurred first, while OS referred to the time from random assignment to death from any cause. Secondary outcomes included disease-controlled rate (DCR), any grade toxicity assessment, rate of side effects leading to drug discontinuation or death, and rate of seven commonly reported adverse events, including hematological (anemia, neutropenia, and thrombocytopenia) and non-hematological (nausea/vomiting, fatigue, diarrhea, and asthenia) adverse events. In addition, we reviewed the published articles about the cost-effectiveness analysis. When updated data for survival was available, the latest data was preferred. If necessary, Parmar’s method was utilized to obtain survival outcomes.

### Quality and risk of bias assessment

Two researchers (L.L.P and J.D.G) independently assessed the risk of bias of the enrolled trials according to the recommendations of the Cochrane Handbook of Systematic Reviews of Interventions (http://handbook.cochrane.org). Disagreements were resolved by discussion.

### Data synthesis and analysis

For PFS and OS, the logarithm of hazard ratio (HR) and their standard error (SE) were pooled into analysis through a Bayesian multiple treatment network meta-analysis with random effects. As for the dichotomous variables, odds ratio (OR) with 95% confidence interval (CI) was applied to calculate. When a network diagram indicated two or more independent loops, only the loop containing “Chemotherapy” was selected for further analysis. The predefined subgroup included PD-L1 expression levels, histology, sex, age, smoking status, ECOG status, and brain metastasis status.

Random effects and consistency model was computed utilizing Markov chain Monte Carlo methods with Gibbs sampling. The algorithm was based on simulations of 50,000 iterations and 20,000 adaptions in each of 4 chains. For a forest plot, “Chemotherapy” was chosen as the common reference comparator. A league table for the survival analysis was presented with the logarithm of HR and their 95% CI. Probability values were summarized and presented with the surface under the cumulative ranking (SUCRA) curve. We also adopted a rank of possibility to provide a hierarchy of treatments concerning both the location and the variance of all relative treatment effects. The SUCRA value would be 0 if treatment is certain to be the worst and 1 if it is certain to be the best. Inconsistency was globally assessed by comparing the fit of consistency and inconsistency models.

All analyses in this article were performed in R-4.3.1 software with the gemtc package version 0.8, while the JAGS version 4.3.0 was utilized for computing a Markov chain. The detailed codes utilized in this analysis also could be obtained from the correspondence author upon request.

## Results

### The search process, study characteristics, and quality assessment

**Fig. 1:**
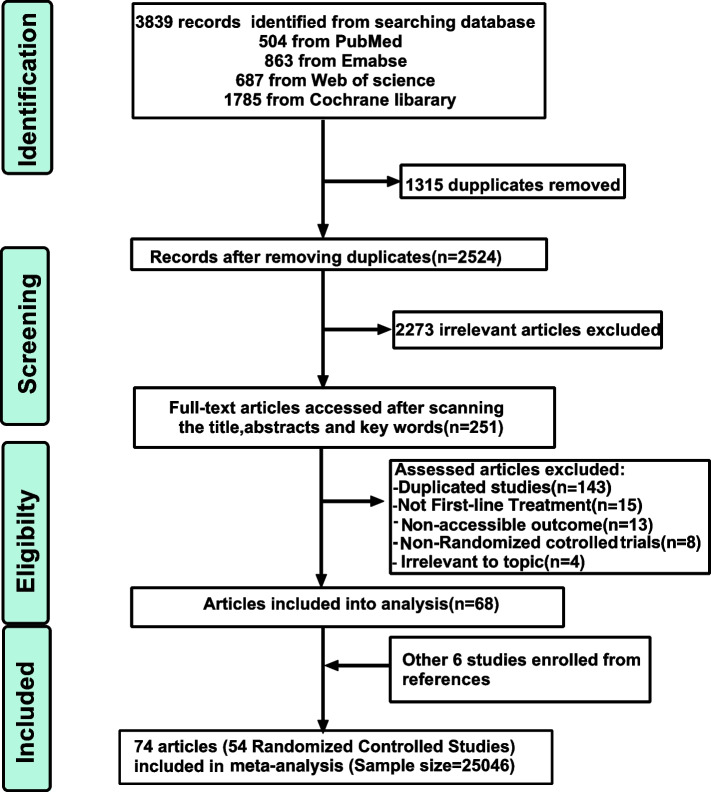
Flow diagram of enrolled studies selection

A total of 3839 records were identified and 2524 records were left to be assessed after removing 1315 duplications. Then, 2273 irrelevant publications were eliminated by skimming their titles, abstracts, and keywords, leaving 251 articles to be considered potentially eligible. 183 articles were further excluded due to duplications (*n* = 143); not first-line treatment (*n* = 15); no accessible data (*n* = 13); non-RCTs (*n* = 8) and irrelevant to topic (*n* = 4). Consequently, 68 articles enrolled into analysis after skimming full-text, and another 6 articles were included by browsing the references. Finally, 74 articles (including 54 studies), published from 2004 to 2021, and with total patients of 25,046 were enrolled into meta-analysis (Fig. 1) [6, 9, 15, 17–87].

**Fig. 2:**
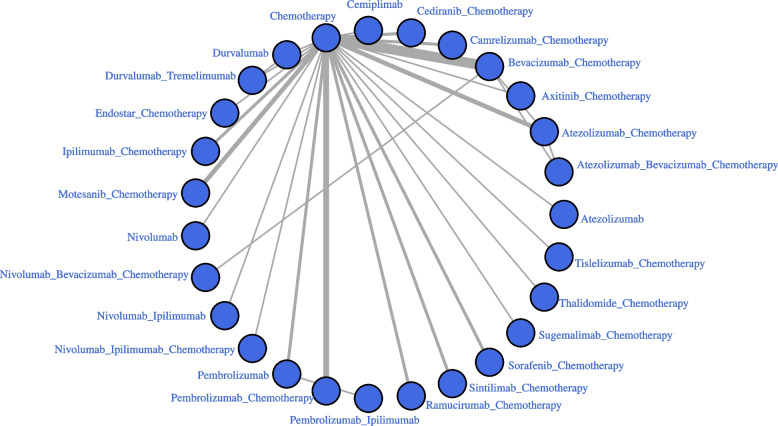
Network evidence for the comparison of all treatment regimens

Table 1 showed the basic characteristics of the enrolled studies and participants. 27 treatment regimens involved:

1. “chemotherapy”;
2. Anti-angiogenetic drugs plus chemotherapy, including “Bevacizumab + Chemotherapy” (Beva-Chemo), “Axitinib + Chemotherapy” (Axintinib-Chemo), “Endostar + Chemotherapy” (Endostar-Chemo), “Cediranib + Chemotherapy” (Cediranib-Chemo), “Motesanib + Chemotherapy” (Mote-Chemo), “Sorafenib + Chemotherapy” (Sora-Chemo), “Ramucirumab + Chemotherapy” (Ramu-Chemo) and “Thalidomide + Chemotherapy” (Thali-Chemo);
3. ICIs monotherapy, including “Atezolizumab” (Atezo), “Pembrolizumab” (Pembro), “Cemiplimab” (Cemip), “Durvalumab” (Durva) and “Nivolumab” (Nivo);
4. ICIs plus chemotherapy, including “Atezolizumab + Chemotherapy” (Atezo-Chemo), “Ipilimumab + Chemotherapy” (Ipili-Chemo), “Camrelizumab + Chemotherapy” (Camre-Chemo), “Pembrolizumab + Chemotherapy” (Pembro-Chemo), “Sugemalimab + Chemotherapy” (Sugema-Chemo), “Sintilimab + Chemotherapy” (Sinti-Chemo) and “Tislelizumab + Chemotherapy” (Tisle-Chemo);
5. ICIs (dual-agent), including “Durvalumab + Tremelimumab” (Dura-Treme); “Nivolumab + Ipilimumab” (Nivo-Ipili) and “Pembrolizumab + Ipilimumab” (Pembro-Ipli) and “Nivolumab + Ipilimumab + Chemotherapy” (Nivo-Ipili-Chemo);
6. Anti-angiogenetic drugs plus ICI-chemo, including “Atezolizumab + Bevacizumab + Chemotherapy” (Atezo-Beva-Chemo) and “Nivolumab + Bevacizumab + Chemotherapy” (Nivo-Beva-Chemo) (Fig. 2).

**Table1:** Basic characteristics of enrolled studies

Study name	Year	Phase	Blind	Stage	Histology	Experiment	Control
AVAiL	2009	III	Double-blinded	IIIB/IV,recurrent	NSCLC	Bevacizumab_Cisplatin_ Gemcitabine	Placebo_Cisplatin_Gemcitabine
BEYOND2016	2015	III	Double-blinded	IIIB/IV,recurrent	Non-squa NSCLC	Bevacizumab _Carboplatin_Paclitaxel	Placebo_Carboplatin_Paclitaxel
BR24	2010	II	Double-blinded	IIIB/IV	NSCLC	Cediranib_ Carboplatin_ Paclitaxel	Placebo_ Carboplatin_Paclitaxel
BR29	2014	III	Double-blinded	IIIB/IV	NSCLC	Cediranib_ Carboplatin_ Paclitaxel	Placebo_ Carboplatin_Paclitaxel
CameL	2020	III	Open-label	IIIB/IV	Non-squa NSCLC	Camrelizumab_Carboplatin_ Pemetrexed	Carboplatin_Pemetrexed
CameL-sq	2021	III	Double blind	IIIB/IV	Squa NSCLC	Camrelizumab _Carboplatin_ Paclitaxel	Placebo _Carboplatin_Paclitaxel
Chandra P Belani2014	2014	II	Open-lable	IIIB/IV,recurrent	Non-squa NSCLC	Axitinib_ Cisplatin_ Pemetrexed	Cisplatin_Pemetrexed
CheckMate 026	2017	III	open-label	IV,recurrent	NSCLC	Nivolumab	Chemotherapy
CheckMate 227	2019	III	open-label	IV	NSCLC	Nivolumab _Ipilimumab	Chemotherapy
CheckMate 9LA	2021	III	open-label	IV,recurrent	NSCLC	Nivolumab_ Ipilimumab_ Chemotherapy	chemotherapy
David H. Johnson 2004	2004	II	Open-lable	IIIB/IV,recurrent	NSCLC	Bevacizumab_Carboplatin_ Paclitaxel	Carboplatin _Paclitaxel
DENG Tao2014	2014	-	-	III/IV	NSCLC	Bevacizumab _ Cisplatin_ Pemetrexed	Cisplatin_Pemetrexed
E4599	2006	III	—	IIIB/IV,recurrent	NSCLC	Bevacizumab_Carboplatin_Paclitaxel	Carboplatin_Paclitaxel
Empower-lung1	2020	III	Open-lable	III/IV	NSCLC	Cemiplimab	Chemotherapy
ERACLE	2015	III	Not-mentioned	IIIB/IV	Non-squas NSCLC	Bevacizumab_Carboplatin_Paclitaxel	Cisplatin_Pemetrexed
ESCAPE	2010	III	-	IIIB/IV	NSCLC	Sorafenib _Carboplatin_Paclitaxel	Placebo _Carboplatin_Paclitaxel
GEMSTONE-302	2020	III	Double-blinded	IV	NSCLC	Sugemalimab _Chemotherapy	Carboplain_Paclitiaxel–SQ;Carbooplatin_Pemetrexed—NSQ
Govindan2017	2017	III	Double blind	IV,recurrent	Squa NSCLC	Ipilimumab _Carboplatin_Paclitaxel	Placebo_Carboplatin_Paclitaxel
HANB2011	2011		Double-blinded	IIIB ~ IV	NSCLC	Endostar_Carboplatin_Paclitaxel	Carboplatin_Paclitaxel
HANL2009	2009					Endostar TXT_DDP	TXT_DDP
Impower110	2020	III	open-label	IV	NSCLC	Atezolizumab	Chemotherapy
IMpower130	2019	III	open-label	IV	Non-squa NSCLC	Atezolizumab_Carboplatin_ Nab-paclitaxel	Carboplatin_ Nab-paclitaxel
IMpower131	2020	III	open-label	IV	Squa NSCLC	Atezolizumab _Carboplatin_ Paclitaxel/Nab-paclitaxel	Carboplatin_Nab-paclitaxel
IMpower132	2020	III	open-label	IV	Non-squa NSCLC	Atezolizumab _Cisplatin/ Carboplatin_ Pemetrexed	Cisplatin/Carboplatin_Pemetrexed
IMpower150	2018	III	open-label	IV,recurrent	Non-squa NSCLC	Atezolizumab_Carboplatin_Paclitaxel; Atezolizumab_Bevacizumab_Carboplatin _ Paclitaxel	Bevacizumab_Carboplatin_Paclitaxel
JO19907	2012	II	Open-lable	IIIB/IV,recurrent	Non-squa NSCLC	Bevacizumab_Carboplatin_Paclitaxel	Carboplatin_Paclitaxel
JSLCG-001	2019	III	Open-lable	IIIB/IV	Squa NSCLC	Endostar_Cisplatin_Docetaxel	Cisplatin_Docetaxel
KEYNOTE-021G	2020	II	open-label	IIIB to IV	Non-squa NSCLC	Pembrolizumab_Carboplatin_Pemetrexed	Carboplatin_Pemetrexed
KEYNOTE-024	2016	III	open-label	IV	NSCLC	Pembrolizumab	Chemotherapy
KEYNOTE-042	2018	III	open-label	III/IV	NSCLC	Pembrolizumab	Carboplatin_ paclitaxel/pemetrexed
KEYNOTE-042China	2020	III	open-label	III/IV	NSCLC	Pembrolizumab	Carboplatin_ paclitaxel/pemetrexed
KEYNOTE-189	2018	III	Double blind	IV	Non-squaNSCLC	Pembrolizumab_Carboplatin/Cisplatin_Pemetrexed	Placebo_Carboplatin/Cisplatin _Pemetrexed
KEYNOTE-407	2020	III	Double blind	IV	Squa NSCLC	Pembrolizumab_Carboplatin_Paclitaxel/Nab-paclitaxel	Placebo_Carboplatin_Paclitaxel /Nab-paclitaxel
Keynote-407 China Extension	2019	III	Double blind	IV	Squa NSCLC	Pembrolizumab_Carboplatin_Paclitaxel /Nab-paclitaxel	Placebo _ Carboplatin_Paclitaxel /Nab-paclitaxel
Keynote-598	2021	III	Double-blinded	IV	NSCLC	Pembrolizumab_Ipilimumab	Pembrolizumab
LOGIK1201	2019	II		IIIB/IV,recurrent	Non-squamous NSCLC	Bevacizumab_Pemetrexed	Pemetrexed
Luis G2012	2012	III	Double-blinded	IIIB-IV	Non-squamous NSCLC	Sorafenib_Cisplatin_Gemcitabine	Placebo_Cisplatin_Gemcitabine
Lynch2012	2012	II	Double blind	IIIB to IV	NSCLC	Ipilimumab_Carboplatin_Paclitaxel	Placebo _Carboplatin_Paclitaxel
MONET1-NSQ	2012	III	Double-blinded	IIIB/IV	Non-squaNSCLC	Motesanib_Carboplatin_Paclitaxel	Carboplatin_Paclitaxel
MONET1-SQ	2014	III	Double-blinded	IIIB/IV,recurrent	Squa NSCLC	Motesanib_Carboplatin_Paclitaxel	Carboplatin_Paclitaxel
MONET-A	2017	III	Double-blinded	IV,recurrent	Non-squamous NSCLC	Motesanib_Carboplatin_Paclitaxel	Placebo_Carboplatin_Paclitaxel
Murakami2010	2010	II	Open-lable	advanced,recurrent	Non-squaNSCLC	Bevacizumab_Cisplatin_Pemetrexed	Cisplatin_Pemetrexed
MYSTIC	2020	III	Open label	IV	NSCLC	Durvalumab;Durvalumab_Tremelimumab	Chemotherapy
ONO-4538–52/TASUKI-52	2020	III	Double-blind	IIIB/ IV	Non-squaNSCLC	Nivolumab_Bevacizumab_ Carboplatin _Paclitaxel	Placebo_Bevacizumab_Carboplatin _Paclitaxel
ORIENT-11	2020	III	Double-blind	IIIB/IV	Non-squamous NSCLC	Sintilimab_Platinum_Pemetrexed	Placebo_Platinum_Pemetrexed
ORIENT-12	2020	III	Double-blind	IIIB to IV	Squa NSCLC	Sintilimab_Cisplatin/Carboplatin_Gemcitabine	Placebo_Cisplatin/Carboplatin_Gemcitabine
Prounce	2015	III	Open-label	IV	Non-squaNSCLC	Bevacizumab_Carboplatin_Paclitaxel	Carboplatin_Paclitaxel
Qun Chen2017	2017	—	—	—	NSCLC	Endostar_Gemcitabine	Gemcitabine
RATIONALE304	2020	III	Open-label	IIIB to IV	Non-squaNSCLC	Tislelizumab _Carboplatin/Cisplatin_Pemetrexed	Carboplatin/Cisplatin_ Pemetrexed
RATIONALE307	2020	III	Open-label	IIIB to IV	Squa NSCLC	Tislelizumab_Carboplatin_Paclitaxel/Nab-paclitaxel	Carboplatin_Paclitaxel
Robert C. Doebele2015	2015	II	Open-label	IV	Non-squaNSCLC	Ramucirumab_Carboplatin /Cisplatin_Pemetrexed	Carboplatin/Cisplatin_ Pemetrexed
S. Thomas2018	2018	II	Open-label	IV	Squa NSCLC	Ramucirumab_Carboplatin /Cisplatin_Gemcitabine	Carboplatin/Cisplatin_Gemcitabine
Siow Ming Lee2009	2009	III	Double-blinded	IIIB/IV	NSCLC	Thalidomide_Carboplatin_Gemcitabine	Placebo_Carboplatin_Gemcitabine
Wang2006	2006	III	Double-blinded	IIIA/IIB/IV	NSCLC	Endostar_Cisplatin_Vinorelbine	Placebo_Cisplatin_Vinorelbine
Xin Zhao 2012	2012	II	Open-label	IIIB/IV	NSCLC	Endostar_Cisplatin_Gemcitabine	Cisplatin_Gemcitabine

AVAiL	345VS.351VS.347	65%VS.62%VS.64%	57(26–81)VS.59(20–83)VS.59(29–83)	—	38%VS.41%VS.41%	—	No
BEYOND2016	138VS.138	54%VS.56%	57(30–75)VS.56(23–74)	50%VS.44%	25%VS.20%	0%vs.0%	Unclear
BR24	126VS.125	58%VS.59%	60(36–77)VS.58(39–81)	—	21%VS.29%	4%VS.4%	Unclear
BR29	153VS.153	55% VS. 54%	63(23–85) VS. 62(32–77)	86%VS. 84%	25%VS. 28%	—	Unclear
CameL	205VS 207	71% VS. 72%	59 (54–64) VS. 61 (53–65)	62%VS. 63%	23% vs. 17%	5% VS. 2%	Yes
CameL-sq	193VS 196	92.7%VS.91.8%	64(34–74) VS. 62(34–74)	83.9%VS.80.1%	19.7%VS.21.9%	Not mention	Yes
Chandra P Belani2014	55VS.58VS.57	62%VS64%VS65%	62 (30–77)VS. 62 (35–83)VS. 59 (42–76)	73%VS.84%VS.79%	45%VS.43%VS.47%	Not-mentioned	No
CheckMate 026	271VS.270	68% VS. 55%	63(32–89)VS.65(29–87)	88%VS. 87%	31% VS. 34%	12% VS. 13%	Yes
CheckMate 227	583VS.583	67.4% VS. 66.0%	64 (26–87)VS. 64 (29–87)	85.2%VS.85.6%	35% VS. 32.8%	-	No
CheckMate 9LA	361VS 358	70% VS. 70%	65(59–70) VS. 65(58–70)	87%VS. 86%	31%vs.31%	18% VS. 16%	No
David H. Johnson2004	32VS.35 VS.32	62.5% VS. 45.7% VS.75%			46.9% VS. 50.0% VS.54.3%		Unclear
DENG Tao2014	120VS. 120	82% VS. 84%	59.3 ± 7.3 VS. 58.8 ± 6.9	Not-mentioned	Not-mentioned	Not-mentioned	Unclear
E4599	417VS.433	50% VS. 58%	≥ 65y(42% VS. 44%)	Not-mentioned	40% VS. 40%	Not-mentioned	Unclear
Empower-lung1	356VS.354	87.6%VS.83.1%	63.0(31.0–79.0)VS.64.0(40.0–84.0)	Not-mentioned	27%VS.27.1%	12.4%VS.11.0%	Yes
ERACLE	58vs.60	70%VS.78%	60 (35–72)VS 62 (41–71)	70%VS.60%	78%VS.79%	Not-mentioned	Unclear
ESCAPE	464VS.462	63% VS. 62%	62(34–86) VS. 63(34–82)	84%VS. 86%	41% VS. 41%	OVS.0	Unclear
GEMSTONE-302	320VS.159	79.4% VS. 81.1%	62.0(29–75) VS.64.0(36–75)	72.5%VS.74.8%	18.4% VS. 15.7%	15.6% VS. 10.7%	Unclear
Govindan2017	388VS.361	84% VS 85%	64(28–84) VS.64(28–85)	87%VS. 88%	35%vs.34%	0 VS 0	Unclear
HANB2011	63VS.63	—	—	—	—	—	Unclear
HANL2009	37VS.31	—	—	—	—	–	Unclear
Impower110	277VS. 277	70.8% VS. 69.7%	64(30–81)VS. 65 (30–87)	86.6%VS.87.3%	35.0%VS.36.8%	No mention	No
IMpower130	483VS. 240	57% VS 58%	64 (18–86)VS. 65 (38–85)	87% VS 92%	42%VS.39%	No mention	Yes
IMpower131	338VS. 343VS .340	82.2% VS 81.6% VS 81.5%	66 (43–85)VS. 65 (23–83)VS. 65 (38–86)	91.1% VS 90.7%VS.92.9%	32.2%vs.33.5%VS.32.4%	No mention	No
IMpower132	292VS. 286	65.8% VS 67.1%	64.0 (31–85)VS. 63.0 (33–83)	87.3% VS 89.5%	43.2%vs.40.1%	No mention	No
IMpower150	400VS.400VS.402	60%VS.59.8%	63 (31 − 89)VS. 63 (31 − 90)	79.5%VS.80.8%	40.1%VS.45.1%	No-mention	No
JO19907	121 VS. 59	64% VS.64%	61 (34–74)VS. 60 (38–73)	69% VS.68%	51% VS. 49%	0VS.0	Unclear
JSLCG-001	94 VS. 94	—	—	—	—	—	Unclear
KEYNOTE-021G	60 VS 63	37% VS. 41%	62.5(40–77)VS.66.0(37–80)	75% VS. 86%	40%VS.46%	20% VS. 11%	Yes
KEYNOTE-024	154VS. 151	59.7% VS 62.9%	64.5 (33–90)VS. 66.0 (38–85)	96.8% VS. 87.4%	35.1%vs.35.1%	11.7% VS.6.6%	Unclear
KEYNOTE-042	637VS.637	71%VS.71%	63.0 (57.0–69.0) VS.63.0 (57.0–69.0)	78%VS.78%	31%VS.30%	5%VS.5%	No
KEYNOTE-042China	—	—	—	—	—	—	No
KEYNOTE-189	410VS.206	62.0% VS. 52.9%	65.0 (34.0–84.0)VS. 63.5 (34.0–84.0)	88.3% VS. 87.9%	45.4% VS. 38.8%	17.8% VS. 17.0%	Yes
KEYNOTE-407	278VS.281	79.1% VS. 83.6%	65.0 (29‒87)VS. 65.0 (36‒88)	92.1% VS.93.2%	26.3%VS.32.0%	7.2% VS. 8.2%	Yes
Keynote-407 China Extension	65vs.60	—	—	—	—	—	—
Keynote-598	284VS.284	71.1%VS.67.3%	64 (35–85)VS.65 (35–85)	89.8%VS.91.2%	35.6%VS.36.6%	10.9%VS.10.2%	Unclear
LOGIK1201	20 VS. 20	60% VS.55%	77.5(75–82)VS.78.5(75–83)	60% VS. 50%	25% VS. 30%	Not-mentioned	Unclear
Luis G2012	385VS.387	59.2% VS. 63.3%	59(28–81) VS. 58(22–77)	72.6% VS. 74.7%	37.9% VS. 37.0%	0VS.0	Unclear
Lynch2012	70vs.68vs.66	76% VS. 72% VS. 74%	59(36–82) VS. 61(36–88) VS. 62(36–82)	Not-mention	27% VS 37% VS 23%	Not mention	Unclear
MONET1-NSQ	541VS.549	62% VS. 61%	60.0 (23–87)VS. 60.0 (21–84)	72% VS. 72%	35% VS.38%	Not-mentioned	Unclear
MONET1-SQ	182VS.178	80% VS. 84%	62.0 (31─79)VS. 59.5 (32─81)	84% VS. 89%	35% VS. 37%	5% VS. 5%	Unclear
MONET-A	197VS.204	71.6% VS. 72.1%	65 (59, 70)VS. 64 (58, 69)	75.1% VS. 68.6%	47.2% VS. 43.6%	Not-mentioned	Unclear
Murakami2010	121.VS. 59	64% VS.64%	61 VS. 60	Not-mentioned	51% vs. 49%	0VS.0	Unclear
MYSTIC	163VS. 163VS.162	69.3%VS.72.4% VS.65.4%	64.0 (32–84)VS. 65.0 (34–87) VS64.5 (35–85)	85.3% VS.84.7% VS.87%	35.0% VS.39.9% VS. 43.2%	Not-mentioned	Unclear
ONO-4538–52/TASUKI-52	237VS.275	—	—	—	—	—	Unclear
ORIENT-11	266VS.131	76.7% VS. 75.6%	61 (30, 75)VS 61 (35, 75)	64.3% VS. 66.4%	28.6% VS. 26.0%	Not mention	Yes
ORIENT-12	179VS.178	91.1% VS. 92.1%	64 (39–75)VS. 62 (33–75)	86.6% VS. 82.6%	16.8% VS. 12.4%	Not mention	Yes
Prounce	179VS.182	58.1% VS. 57.7%	65.4 (41.2–86.2)VS.65.8 (38.4–84.1)	90.1% VS. 96.1%	46.7% VS. 46.9%	12.6% VS.17.9%	Unclear
Qun Chen2017	62 VS. 56	—	—	—	—	––	Unclear
RATIONALE304	223VS 111	75.3% VS 71.2%	60(27,75) VS. 61(25,74)	65.9% VS. 59.5%	24.2% VS 21.6%	4.9% VS. 6.3%	Yes
RATIONALE307	120VS.119 VS 121	89.2%VS94.1% VS 91.7%	60 (41–74)VS. 63 (38–74)VS. 62 (34–74)	80.0% VS 89.9% VS 81.0%	25.8% VS. 18.5% VS 26.4%	1.7% VS. 2.5% VS. 0.8%	Yes
Robert C. Doebele2015	69 VS.71	52.2% VS. 63.4%	≥ 65y(46.4% VS. 47.9%)	22.5% VS. 15.9%	-	Not-mentioned	Unclear
S. Thomas2018	71 VS. 69	—	—	—	—	—	Unclear
Siow Ming Lee2009	372VS.350	65% VS. 64%	63(35–84) VS. 62(33–84)	Not-mentioned	30% VS. 31%	Not-mentioned	Unclear
Wang2006	230VS.117	—	—	—	—	—	Unclear
Xin Zhao2012	33 VS. 36	63.64%VS.69.44%	61(37–73)VS. 60 (35–72)	Not-mentioned	12.12% VS. 13.89%	Not-mentioned	No

*NSCLC* Non-small cell lung cancer

Based on the Cochrane Risk of Bias Tool, 28 studies had an overall high risk of bias, 9 studies had an overall low risk of bias while the other 17 studies had an unclear risk of bias (Supplementary Fig. 1A-B). The domain of “blinding of participants and personnel” contributed to the biggest sources of high risk due to the open-label design of 26 enrolled studies.

### Primary outcome—PFS

**Fig. 3:**
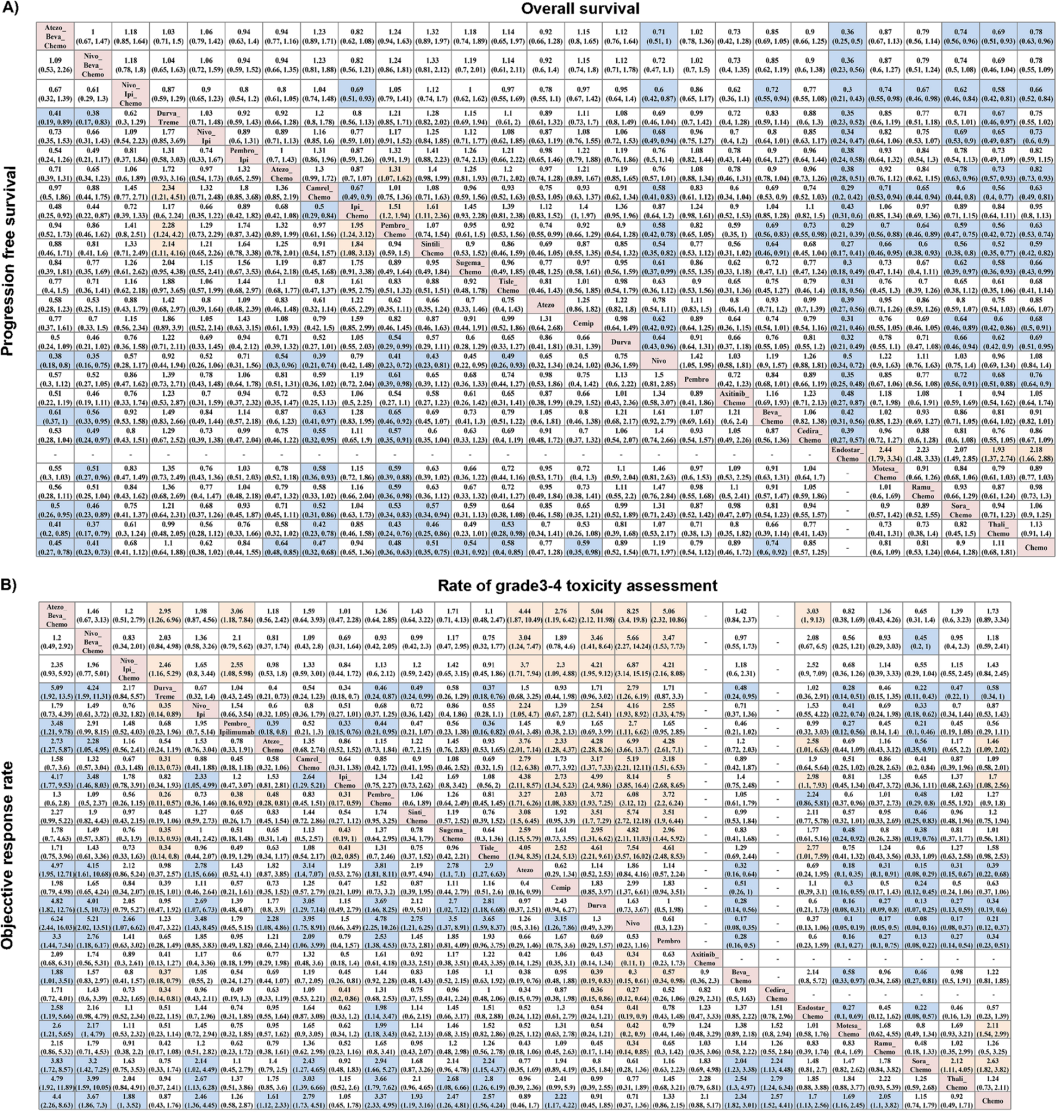
Pooled estimates of the network meta-analysis. **A** Pooled hazard ratios (95% credible intervals) for progression-free survival(lower triangle) and overall survival(upper triangle). **B** Pooled odds ratios (95% credible intervals) for objective response rate(lower triangle) and rate of grade3-4 toxicity assessment(upper triangle)

NMA involved 27 treatment regimens except Endostar-Chemo for PFS analysis (Fig. 3A). Atezo-Beva-Chemo and Nivo-Beva-Chemo had a better survival benefit compared with Beva-Chemo (HR = 0.61, 95% CI: 0.37–1; HR = 0.56, 95% CI: 0.33–0.95, respectively). The trend favored Atezo-Beva-Chemo to Atezo-Chemo in PFS with no statistical significance (HR = 0.71, 95%CI: 0.39–1.31). Camre-Chemo significantly prolonged the PFS compared with Ipili-Chemo (HR = 0.5, 95%CI: 0.29–0.84) and Beva-Chemo (HR = 0.65, 95%CI: 0.46–0.9). In addition, Pembro-Chemo had a significant survival benefit compared to anti-angio-chemo including Beva-Chemo (HR = 0.65, 95%CI: 0.46–0.92), Ramu-Chemo (HR = 0.59, 95%CI: 0.36–0.98) and other regimens. However, Durva-Treme was significantly inferior to the Camre-Chemo (HR = 2.34, 95%CI: 1.21–4.51), Pembro-Chemo (HR = 2.28, 95%CI: 1.24–4.2) and Sinti-Chemo (HR = 2.14, 95%CI: 1.11–4.16) in PFS. Ipili-Chemo was also inferior to the Pembro-Chemo (HR = 1.95, 95%CI: 1.24–3.12) and Sinti-Chemo (HR = 1.84, 95%CI: 1.08–3.13).

### Primary outcome—OS

NMA involved all 27 treatment regimens for OS analysis (Fig. 3A**)**. Atezo-Beva-Chemo performed a divorced trend to Atezo-Chemo regarding OS (HR = 0.94, 95%CI: 0.77–1.16). Camre-Chemo and Sinti-Chemo presented a statistically significant OS benefit compared with the Ipili-Chemo (HR = 0.67, 95%CI: 0.49–0.9; HR = 0.62, 95%CI:0.42–0.90, respectively). Atezo-Chemo and Ipili-Chemo was inferior to Pembro-Chemo in providing OS benefit (HR = 1.31, 95%CI: 1.07–1.62; HR = 1.51, 95%CI: 1.2–1.94). In addition, Nivo-Ipili-Chemo, Pembro-Chemo and Sinti-Chemo obtained a better survival benefit than Beva-Chemo with HR of 0.72 (95%CI: 0.55–0.94), 0.69 (95%CI: 0.56–0.83), and 0.64 (95%CI: 0.46–0.91), respectively. Noteworthy, almost all regimens were superior to Endostar-Chemo in improving OS.

### Primary outcome—ORR

NMA involved 27 treatment regimens for ORR analysis (Fig. 3B). Atezo-Beva-Chemo improved ORR significantly compared to the Atezo-Chemo (OR = 2.73, 95%CI: 1.27–5.87) and Bevi-Chemo (OR = 1.88, 95%CI: 1.01- 3.51). Likewise, Nivo-Beva-Chemo also yield a significantly higher ORR than Durva-Chemo (OR = 4.24, 95%CI: 1.59–11.31), and Atezo-Chemo (OR = 2.28, 95%CI: 1.05–4.95) (OR = 3.48,95%CI: 1.46–8.03). However, ORR was significantly lower in the Durva-Treme group comparing to ICI-chemo regimens, including with ORR of 0.31 to Camre-Chemo (95%CI: 0.13–0.73) and 0.26 to Pembro-Chemo (95%CI: 0.11–0.57). ORR was lower in the Pembor-Ipi group comparing to the Pembro-Chemo group (OR = 0.38, 95%CI: 0.16–0.92). Similarly, ORR was significantly lower in the Ipili-Chemo group comparing to Pembro-Chemo (OR = 0.31, 95%CI: 0.17–0.59) and Tisle-Chemo (OR = 0.41, 95%CI: 0.20–0.85). In addition, ORR was significantly improved in the Camre-Chemo group comparing to the Ipili-Chemo group (OR = 2.64, 95%CI: 1.29–5.21). ORR was significantly lower in Atezo-Chemo group comparing to the Pembro-Chemo group (OR = 0.48, 95%CI: 0.28–0.81).

### Primary outcome— rate of grade3-4 toxicity assessment

NMA involved 27 treatment regimens for rate of grade3-4 toxicity assessment (Fig. 3B). No significant difference was observed between Atezo-Beva-Chemo and Atezo-Chemo (OR = 1.18, 95%CI: 0.56–2.42) in the rate of grade3-4 toxicity. Meanwhile, Durva-Treme served lower rate of grade3-4 toxicity comparing to Pembro-Chemo (OR = 0.46, 95%CI: 0.24–0.87), Sinti-Chemo (OR = 0.49, 95%CI: 0.24–0.99) and Tisle-Chemo (OR = 0.37, 95%CI: 0.18–0.76). Likewise, Pembro-Ipili served lower than Ipili-Chemo (OR = 0.33, 95%CI: 0.15–0.76) and Pembro-Chemo (OR = 0.44, 95%CI: 0.21–0.95).

### Subgroup analysis of PFS and OS stratified by PD-L1 expression level

**Fig. 4:**
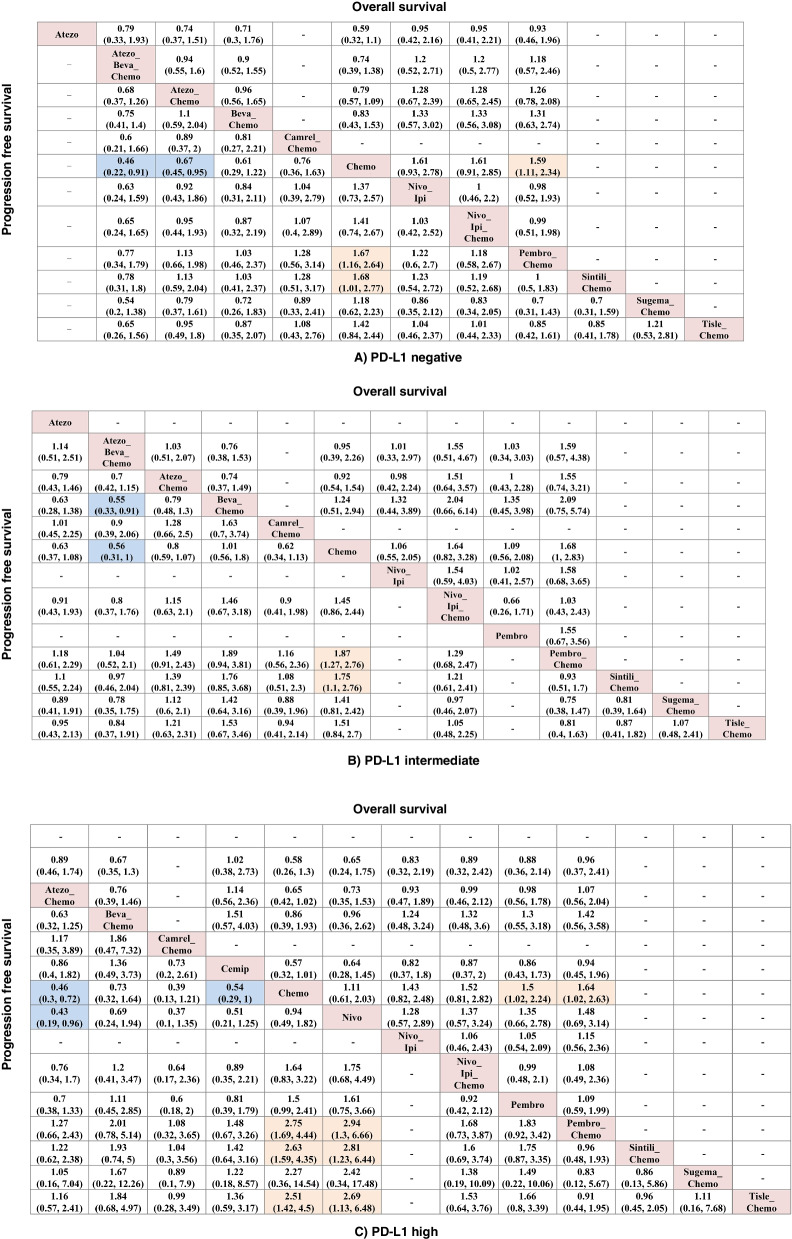
Progression-free survival (lower triangle) and overall survival (upper triangle) comparison profile for advanced NSCLC under subgroup analysis stratified by PDL-1 expression status. **A** PD-L1 negative. **B** PD-L1 intermediate. **C** PD-L1 high

1. For PD-L1-negative patients, NMA involved 11 treatment regimens for PFS and 7 treatment regimens for OS (Fig. 4A). Atezo-Beva-Chemo and Atezolizumab alone appeared to obtain more survival benefit than Chemotherapy alone in prolonging PFS with HR of 0.46 (95%CI: 0.22–0.91) and 0.67 (95%CI: 0.45–0.95), respectively. In addition, the PFS of both Pembro-Chemo and Sinti-Chemo was obtain significantly higher PFS benefits than Chemotherapy alone with HR of 0.60 (95%CI: 0.38–0.86) and 0.60 (95%CI: 0.36–0.99), respectively. Meanwhile, Pembro-Chemo significantly pronged OS compared with Chemotherapy (HR = 0.63, 95%CI: 0.43–0.90)  (Fig. 4A).
2. For PD-L1-intermediate patients, NMA involved 10 treatment regimens for PFS and 7 treatment regimens for OS (Fig. 4B). Atezo-Beva-Chemo yielded a better PFS benefit comparing to Beva-Chemo (HR = 0.55, 95%CI: 0.33–0.91) and “Chemotherapy” alone (HR = 0.56, 95%CI: 0.31–1). In addition, Pembro-Chemo and Sinti-Chemo yielded a better PFS benefit comparing to Chemotherapy alone (HR = 0.53, 95%CI: 0.36–0.79; HR = 0.57, 95%CI: 0.36–0.90). No significant differences in OS were observed among all regimens for PD-L1-intermediate patients.
3. For PD-L1-high patients, NMA involved 13 treatment regimens for PFS and 9 treatment regimens for OS (Fig. 4C). Atezo-Beva-Chemo had a better PFS benefit than Beva-Chemo (HR = 0.33, 95%CI: 0.16–0.67) and “Chemotherapy” (HR = 0.24, 95%CI: 0.11–0.55). Also, Atezo-Chemo could significantly prolong PFS comparing to Chemotherapy alone (HR = 0.46, 95% CI: 0.30–0.72). Cemiplimab appeared to extend PFS versus “Chemotherapy” (HR 0.54, 95%CI 0.29–1). Pembro-Chemo (HR = 0.36, 95% CI: 0.23–0.59), Sinti-Chemo (HR = 0.38, 95%CI: 0.2–0.63) and Tisleli-Chemo (HR = 0.40, 95%CI: 0.22–0.70) had a better PFS benefit comparing to Chemotherapy alone. Pembro-Chemo and “Pembrolizumab” monotherapy yielded better OS benefits than “Chemotherapy” alone (HR = 0.67, 95%CI: 0.45–0.98; HR = 0.61, 95%CI: 0.38–0.98; respectively).

### Subgroup analysis of PFS and OS stratified by other risk factors

Survival analysis was stratified by histology, sex, age, smoking status, ECOG status, and brain metastasis status (Supplementary Fig. 2). For non-squamous NSCLC patients, ICI-chemos including Atezo-Chemo, Pembro-Chemo, Sinti-Chemo, and Beva-Chemo obtained significantly better PFS benefits than Chemotherapy alone with HR of 0.60 (95%CI: 0.36–1.00), 0.50 (95%CI: 0.29–0.87), 0.48 (95%CI:0.22–1.0) and 0.66 (95%CI:0.45–1.0), respectively. Meanwhile, Pembrolizumab, Pembro-Chemo, Sinti-Chemo, Nivol-Ipili-Chemo could significantly extend OS comparing to Chemotherapy alone with HR of 0.58 (95%CI: 0.37–0.92), 0.59 (95%CI: 0.45–0.82), 0.61(95%CI: 0.37–1.0) and 0.69 (95%CI: 0.47–1.0), respectively. However, for squamous advanced NSCLC patients, no regimens could significantly extend PFS except Sugema-Chemo (HR = 0.33, 95%CI: 0.11–1.0). As for subgroup analysis according to other risk factors including sex, age, smoking status, and ECOG status, results were generally consistent with those above-mentioned unselected patients.

### Rank probabilities for primary outcomes

As it was presented in Supplementary Table 1**,** the results of the Bayesian ranking profile were consistent with the pooled analysis using HR and OR. Nivo-Beva-Chemo was most likely to be ranked first for PFS (cumulative probability 37%), Sinti + Chemo for OS (30%), Atezo-Beva-Chemo for ORR (50%), and “Nivolumab” for decreasing rate of grade3-4 toxicity assessment. In contrast, “Nivolumab” was to be ranked worst for PFS (31%). Endostar + Chemotherapy for OS (99%), Nivolumab for ORR (39%), and Sora-Chemo for decreasing rate of grade3-4 toxicity (67%).

**Fig. 5:**
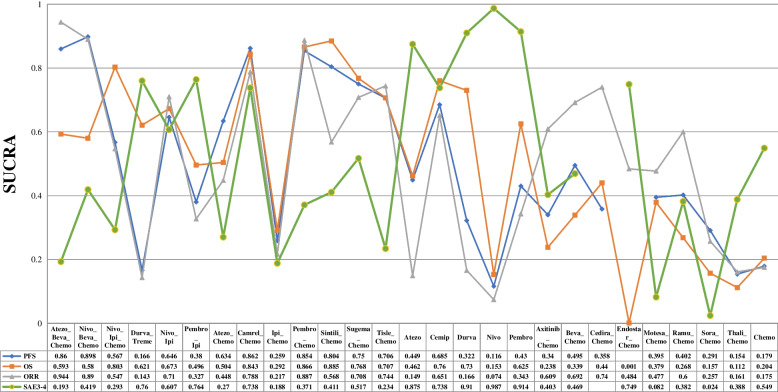
Bayesian ranking profile based on the SUCRA results of progression-free survival, overall survival, objective response rate, and decrement rate of grade 3–4 assessment

Bayesian ranking profile based on SUCRA results was also in line with the HR and OR estimates (Fig. 5). Nivo-Beva-Chemo appeared to have the highest probability of pronging PFS (SUCRA = 0.898), followed by Camre-Chemo (0.862), Atezo-Beva-Chemo (0.86), and Pembro-Chemo (0.854). Concerning the extension of OS, Sinti-Chemo had the highest probability to be the best regimen (0.885), followed by Pembro-Chemo (0.866) and Camre-Chemo (0.843). In terms of ORR, Atezo-Beva-Chemo was most likely to improve ORR (0.944), followed by Nivo-Beva-Chemo (0.89), Pembro-Chemo (0.887) and Camre-Chemo (0.788). However, “Nivolumab” appeared to have the best grade 3–4 safety profile (0.987), followed by “Pembrolizumab” (0.914) and “Durvalumab” (0.910).

### Secondary outcomes—DCR, any grade toxicity assessment, rate of side effects leading to discontinuation and death

NMA involved 20 treatment regimens for DCR, 18 regimens for any grade toxicity assessment, 22 regimens for rate of side effects leading to drug discontinuation, and 19 regimens for rate of side effects leading to death. Bayesian ranking profile of secondary outcomes based on SUCRA was presented in Supplementary Fig. 3**.** Pembro-Chemo had the highest probability for improving DCR (SUCRA = 0.768), followed by Atezo-Chemo (0.746) and Camre-Chemo (0.745). However, as for any grade toxicity assessment, Endostar-Chemotherapy had the best probability for reducing all grade toxicity assessment (0.954), followed by Sinti-Chemo (0.872). In terms of decreasing rate of side effects leading to drug discontinuation, “Durvalumab” had the lowest risk (0.961), followed by Sinti-Chemo (0.887). In addition, Sinti-Chemo had the lowest risk of suffering from death caused by side effects (0.851), followed by “Atezolizumab” (0.709).

### Secondary outcome—specific adverse events

NMA involved 25 regimens for anemia, 24 regimens for neutropenia, 21 regimens for thrombocytopenia, 22 regimens for fatigue, 19 regimens for diarrhea, 25 treatment regimens for nausea/vomiting, and 15 regimens for asthenia. Bayesian ranking profile of specific adverse events based on SUCRA was showed on Supplementary Fig 4**.** Durva-Treme had the lowest risk of anemia (SUCRA = 0.983) and asthenia (0.739). Meanwhile, “Durvalumab” had the lowest risk of thrombocytopenia (0.959). In addition, “Nivolumab” had the lowest risk of fatigue (0.939), while “Cemiplimab” had the lowest risk of diarrhea (0.958), and “Pembrolizumab” had the lowest risk of nausea/vomiting (0.964).

### Secondary outcome—Cost-effectiveness analysis

**Table ****2** summarized the 21 reviewed studies about the cost-effectiveness analysis based on our above-mentioned enrolled studies, including Beyond [65], CheckMate 227 [66], E4599 [67], LOGIK0201 [55], Impower-110 [68, 69], Impower-130 [70, 71], Keynote-021G [72], Keynote-024 [73–76], Keynote-042 [24, 26, 39], Keynote-189 [25, 38], Keynote-407 [38, 77, 78] and Pronounce [40] trials. The quality-adjusted life-year (QALY), incremental cost-effectiveness ratio (ICER), life-years (LY), and total cost were commonly used for effectiveness measures. All reviewed studies except three studies applied sensitivity analysis to deal with the effect of uncertainty in results and their generalization ability. The annual discount rate ranged from 3 to 5%.

**Table 2:** Description of cost-effectiveness study characteristics

First Author	Year	Country	Study	Effectiveness measure	Model	Time period	Sensitity analysis	Discount rate	ICER	QALY(Y)
Xinyan-Li	2018	China	Beyound	QALY,LY,Total cost,ICER	Markov	Ten-years	Yes	3%	B + CP VS. PI + CP:$130,937.09/QALY	1.17;0.83
Bhadhuri-Arjun	2019	Switzerland	Keynote-024	QALY,LY,Total cost,ICER	A partitioned survival model	20 years	Yes	3%	Pembrolizumab(223,324 CHF) vs. chemotherapy(CHF146,264):CHF 77,060 per QALY	3.05;1.71
Guoqiang liu	2021	China	Impower-110	QALY,LY,Total cost,ICER	Adecision-analytic model	10 years	Yes	5%	Atezolizumab VS. Chemotherapy: $168,902.66/QALY	1.31;0.90
Ye Peng	2021	USA	Impower-110	QALY,LY,Total cost,ICER	Markov	-	Yes	3%	Atezolizumab VS. Chemotherapy: $170,730/QALY	2.36;1.08
Zhiguang Yang	2021	China	Impower-130	QALY,LY,Total cost,ICER	Markov	-	Yes	5%	Atezolizumab + Chemotherapy vs. Chemotherapy:$325,328.71 QALY	0.87;0.68
Shen lin	2020	USA	Impower-130	QALY,LY,Total cost,ICER	Markov	10 years	Yes	3%	Atezolizumab + Chemotherapy vs. Chemotherapy:$ 333,199QALY	0.99;0.67
Xu H	2020	China	Keynote-042	QALY,ICER	Markov; partition-survival		Yes		PembrolizumabVS.Chemotherapy(CNY244,495.54/QALY)	—
Insinga-R	2020	Taiwan	Keynote-189 + Keynote-407	ICER,QALY	partition-survival	20 years	No	3%	—	—
Ralph.P.Insinga	2019	US	Keynote-407	ICER,QALY	partition-survival	20 years	Yes	3%	Pembrolizumab + Chemotherapy vs. Chemotherapy:$86,293/QALY	3.86;1.91
H.Loong	2017	Hong-Kong	Keynote-024	ICER,QALY,Total-cost	partition-survival	10 years	Yes	3%	PembrolizumabVS. chemotherapy:HK$865,189(USD110,922)per QALY	0.29(by)
Min-Huang	2017	USA	Keynote-024	QALY,LY,ICER	partition-survival	20 years	Yes	3%	PembrolizumabVS. chemotherapy:$US97,621per QALY	2.60;1.55
Pinheiro-BA	2017	Portugal	Keynote-024	QALY,LY,ICER	partition-survival		Yes	5%	PembrolizumabVS.chemotherapy:64,205$per QALY	1.7(by)
Kexun-Zhou	2019	China	Keynote-042	ICER,QALY,Total-cost	Markov	10 years	Yes	3%	PembrolizumabVS.Chemotherapy($39,404/QALY)	2.16;1.04
Min-Huang	2019	USA	Keynote-042	QALY,LY,ICER	partition-survival	20 years	Yes	3%	PembrolizumabVS.Chemotherapy($130,155/QALY)	1.77;1.28
Ralph.P.Insinga	2018	US	Keynote-189	QALY,LY,Total cost,ICER	partition-survival	20 years	Yes	3%	Pembrolizumab + Chemotherapy vs. Chemotherapy:$104,823/QALY	2.84;1.40
Chouaid,C	2020	France	Keynote-407	QALY,LY,ICER	partition-survival	10 years	Yes	4%	Pembrolizumab + Chemotherapy vs. Chemotherapy:$116,606/QALY	0.46(by)
Tfayli-A	2018	Lebanon	Pronounce	QALY,ICER	Markov	5 years	Yes	—	BevicizumabVS.Carboplatin + Pemetrexed	6.6(by)
Bernardo Goulart	2011	USA	E4599	QALY,ICER,LY	Markov	4 years	Yes	3%	Bevicizumab + Carboplatin + Paclitaxel VS. Placebo + Carboplatin + Paclitaxel $559,609.48	0.66;0.53
P. Travis Courtney	2021	USA	CheckMate 227	QALY,ICER,Total-cost,	Markov	10 years	Yes	3%	Nivolumab-ipilimumab VS.Chemotherapy: $401 700 per QALY	1,68;1.18
Minoru-Fukuda	2019	Japan	LOGIK0201	Total-cost	—	—	No		Bevacizumab + Pemetrexed(3,368,428)VS.Pemetrexed(1,522,008)	
Bestvina, C	2017	USA	Keynote-021G	Total-cost	—	—	No		Carboplatin/pemetrexed/pembrolizumab($618,889)–Carboplatin/pemetrexed($249,972)	—

*QALY* Quality-adjusted life-year, *LY* life-years, *ICER* incremental cost-effectiveness ratio

For Beva-Chemo, ICER was reported to be $130,937.09/QALY (Beyond trial in China) and $559,609.48/QALY (E4599 trial in USA) compared with Chemotherapy alone. Concerning the comparison between Atezolizumab and Chemotherapy alone, ICER was reported to be $168,902.66/QALY in China and $170,730/QALY in the USA (Impower-110 trial). In addition, Atezo-Chemo versus Chemotherapy alone could achieve ICER at $325,328.71/QALY in China and $ 333,199/QALY in the USA (Impower-130 trial). As for Pembrolizumab versus Chemotherapy in Keynote-024 trial, ICER was reported to be $865,189 /QALY (HongKong China), CHF 77,060/QALY(Switzerland), $97,621/QALY (USA) and $64,205/QALY (Portugal). Meanwhile, based on Keynote-042 trial, Pembrolizumab versus “Chemotherapy” could achieve ICER at $39,404/QALY (China) and $130,155/QALY (USA). As for Pembro-Chemo versus Chemotherapy alone, ICER was reported to be $104,823/QALY (Keynote-189 trial in the USA), $116,606/QALY (Keynote-407 trial in France), and $86,293/QALY (Keynote-407 trial in the USA).

### Inconsistency assessment

The fit of the consistency model was similar or even better than that of the inconsistency model (Supplementary Table 2).

## Discussion

Currently, there is no “head-to-head” trial comparing ICI-chemo versus anti-angio-chemo to validate their comparative efficacy and safety. Meanwhile, ICI plus Beva-Chemo holds the potential to obtain better survival benefits but may be at the expense of toxicities. Nevertheless, the true impact of adding anti-angiogenetic agents to ICI-chemo remains inconclusive. The development of ICIs has resulted in a shift in the first-line treatment landscape for NSCLC patients. Constantly increasing new drugs or therapeutic combinations are formally approved. In this study, we enrolled well-designed RCTs and conducted a Bayesian NMA to compare the efficacy, safety, and cost-effectiveness of different first-line treatment regimens for advanced NSCLC patients. The diversity of responses to different regimens and corresponding toxicity concerns were observed in this study.

Optimal therapeutic strategies could delay the occurrence of drug resistance thus potentially redefining the survival outcome. Atezo-Beva-Chemo was the best regimen to improve significantly ORR comparing to Atezo-Chemo and Beva-Chemo. Atezo-Beva-Chemo and Nivo-Beva-Chemo obtained better survival benefits compared with Beva-Chemo. There was a trend for Atezo-Beva-Chemo to perform better than Atezo-Chemo in assessment of PFS (HR = 0.71, 95%CI: 0.39–1.31). A better trend for Atezo-Beva-Chemo compared to Atezo-Chemo in OS was also revealed (HR 0.94, 95%CI 0.77–1.16). Of note, Nivo-Beva-Chemo was most likely to be ranked first for extending PFS (cumulative probability 37%), while Atezo-Beva-Chemo had the highest probability to be ranked first for improving ORR (50%). Our results suggested that the efficacy could be enhanced after adding Bevacizumab to the ICI-chemo. Therapeutically, the immune-suppressive microenvironment could be converted to be immune-permissive through the immunomodulatory effects of antiangiogenic agents, thus improving the capacity of ICIs [13, 14]. Meanwhile, in terms of side effects, no significant difference was observed between ICI-chemo and Atezo-Beva-Chemo or Nivo-Beva-Chemo. Adding bevacizumab into ICI-chemo seemed to provide additional benefits without adding a significant treatment burden. Remarkably, serval ongoing registered trials [88, 89] have been conducted to investigate the clinical benefit of adding anti-angiogenic agents into ICI-chemo in patients with advanced NSCLC.

Meanwhile, Camre-Chemo, Pembro-Chemo, Sinti-Chemo, and Tisle-Chemo also showed advantages over Beva-Chemo in providing PFS benefit. Notably, Sinti-Chemo and Pembro-Chemo showed better OS benefits than Beva-Chemo. Sinti-Chemo ranked best to be the regimen of extending OS benefits. In particular, Endostar-Chemo ranked the worst for OS benefits. The reason of worse efficacy of anti-angio-chemo may be associated with the resistant mechanism and compensatory pathway of angiogenesis in tumor [90]. PD-1 inhibitors in combination with platinum-based chemotherapy hold the potential to prolong patients’ life expectancy. However, our results should be interpreted with caution due to the variety of follow-up periods and post-progression interventions in different treatment groups. Therefore, further mature OS data and head-to-head RCTs were warranted to be performed. In terms of the rate of grade3-4 toxicity, no significant difference was observed between ICI-chemo and anti-angio-chemo. As for cost-effectiveness analysis, it seemed that ICI-chemo yielded more improvement in QALY than Beva-Chemo. In conclusion, ICI-chemo is associated with potentially higher survival and better cost-effectiveness outcomes than anti-angio-chemo with comparable safety profiles.

Noteworthy, Ipili-Chemo (CTLA-4 inhibitors plus chemotherapy) was inferior to Camre-Chemo, Pembro-Chemo and Tisleli-Chemo in improving ORR and extending survival. In comparison with CTLA-4, our results inferred that PD-1/PDL-1 may play a more important role in the immune response for patients with advanced NSCLC. However, there was no significant difference between the PD-1/PDL-1 and CTLA-4 inhibitors monotherapy. Of note, Durva-Treme was significantly inferior to the ICI-Chemo regarding the ORR and PFS benefit, with lower rate of grade3-4 toxicity. It is reasonable to interpret that ICIs (dual agent) monotherapy are not the optimal therapeutic methods in obtaining survival benefit despite their superior safety profiles. Nevertheless, Nivo-Ipili-Chemo obtained a better survival benefit than Beva-Chemo. Anti-PD-1 and anti-CTLA-4 antibody are ICIs with distinct but complementary mechanisms of action. Consequently, ICIs (dual agent), especially PD-1 plus CTLA4 inhibitors, in combination with chemotherapy may represent the focus in designing the future clinical trials.

In addition, Pembro-Chemo could significantly perform better than Atezo-Chemo in ORR at this study. Likewise, our results suggested that Sinti-Chemo and Pembro-Chemo showed more OS benefits than Atezo-Chemo. A potential biological explanation is that Pembrolizumab and Sintilimab (PD-1 inhibitors) rather than Atezolimumab (PD-L1 inhibitors) block the binding between PD-1 and corresponding ligands PD-L2, which is estimated to be 2–6 folds stronger than the affinity of PD-1 binding to PD-L1 [88]. However, no statistically significant difference was observed between Pembrolizumab and Atezolimumab monotherapy. Although this could be partly explained by the imbalance in the basic characteristics of the enrolled studies, the underlying mechanism still warranted further exploration. Given the promising results of PD-1 inhibitors plus chemotherapy, further research is supposed to shed light on the combination of PD-1 inhibitors plus chemotherapy and anti-angiogenetic agents, e.g. Pembrolizumab + Bevacizumab + Chemotehrapy.

The diversity of responses to ICIs has raised the questions about how to better tailor the treatment strategy and choose the best-targeted population. The PD-L1 expression status is a potential biomarker [89]. For PD-L1 negative patients, Atezol-Beva-Chemo, Pembro-Chemo and Sinti-Chemo obtained more survival benefit than Chemotherapy alone in prolonging PFS. Moreover, Pembro-Chemo showed advantages over Chemotherapy alone in the extension of OS. Chemotherapy induces recruitment of CD8 + cells and provides an appropriate binding site for ICIs [7]. For PD-L1 intermediate patients, Atezoli-Beva-Chemo yielded the best survival benefit compared with Beva-Chemo and Chemotherapy alone. In addition, Pembro-Chemo and Sinti-Chemo serves better than Chemotherapy alone in these settings. For PD-L1 high NSCLC patients, Atezo-Beva-Chemo had significantly longer PFS than Beva-Chemo and Chemotherapy. Also, Atezo-Chemo, Pembro-Chemo, Sinti-Chemo and Tisle-Chemo could significantly prolong PFS compared with Chemotherapy alone. Pembro-Chemo yielded a better OS survival than Chemotherapy alone. These results were generally consistent with the above-mentioned results in the unselected patients and previously reported results. Of note, Atezol-Beva-Chemo, Pembro-Chemo and Sinti-Chemo obtained more survival benefit than Chemotherapy alone in prolonging PFS, irrespective off the PD-L1 expression level. Furthermore, Pembro-Chemo is the only regimen to extend OS for PD-L1 negative or PD-L1 high patients. Nevertheless, given the relatively limited data of targeted treatment regimens, the variety of detection methods or predefined thresholds of PD-L1 expression, our results should be interpreted with caution. Some misclassification bias could lead to underestimation or overestimation of treatment results in various PD-L1 cohorts.

The immune microenvironment of squamous NSCLC patients presents different [91–93]. For squamous advanced NSCLC patients, we found that except “Suge-Chemo”, no regimens could significantly extend PFS. However, results in the non-squamous NSCLC patients’ group were generally in line with the unselected group. This distinct result in different pathological types highlights the urgent need for further exploration of the mechanism behind the tumor microenvironment.

The toxicity and adverse events are the major concern when prescribing the combined regimens. Consistent with previously studies, our results showed that ICIs monotherapy had the best safety profile compared with chemotherapy and other regimens [16, 33]. Our results provided further evidence supporting the toxicity assessment of different combined regimens. Compared with ICIs monotherapy, our results suggested that better efficacy may occur in the combined regimens (ICIs plus chemotherapy) with increasing side effects. As for the cost-effectiveness analysis, ICIs monotherapy or ICI-chemo yielded more improvement in QALY than Beva-Chemo. In addition, Pembro-Chemo appeared to obtain more QALY benefits than Atezo-Chemo. Due to the differences in the study horizons and the costs of expenditure, our results warrant further confirmation.

To the best of our knowledge, the present study presents the most comprehensive NMA to compare ICI-chemo with anti-angio-chemo. Although a published indirect-comparison has demonstrated that ICI-chemo is superior to Beva-Chemo in first-line treatment for non-squamous NSCLC, they failed to observe the respective characteristics of different therapeutic regimens [94]. Another highlight of our analysis is to answer an important question of whether anti-angiogenetic agents plus ICI-chemo could provide additional benefits or change the safety profile compared with ICI-chemo for the first time. A large-scale number of subjects involved in a meta-analysis is critically important to reduce the statistical errors. Concerning the first-line treatment landscape for advanced NSCLC, the current study enrolled the largest scale of patients so far.

There were some limitations in this study. Firstly, several included studies were conference abstracts, where we could not obtain all data and assess the risk of bias. Secondly, the original data was limited since some trials were ongoing. Thirdly, uniform methods in assessment of PD-L1 expression status exerted a negative effect on the subgroup analysis stratified by PD-L1 expression level. Last but not least, various follow-up periods and trial designs of enrolled studies imposed the heterogeneity of the present study.

In summary, our results suggest that ICI-chemo is associated with better survival benefits and cost-effectiveness outcomes than anti-angio-ICI with comparable safety profiles. Adding bevacizumab to ICI-chemo seemed to provide additional therapeutic benefits without extra treatment burden. Atezol-Beva-Chemo, Pembro-Chemo and Sinti-Chemo could obtain more survival benefits than Chemotherapy alone in prolonging PFS irrespective off the PD-L1 expression level. Our findings could supplement the current standard of care and lead the design of the future clinical trials in the first-line treatment of patients with advanced NSCLC.

## Supplementary Information

**Additional file 1.**

**Additional file 2.**

**Additional file 3: Supplementary Figure 1.** Risk of bias assessment. A Risk of bias graph. B Risk of bias summary.

**Additional file 4: Supplementary Figure 2. **Progression-free survival and overall survival comparison profile for advanced NSCLC under subgroup analysis stratified by histology, sex, age, smoking status, ECOG status, and brain metastasis or not.

**Additional file 5: Supplementary Table 1.** Bayesian ranking results of network meta-analysis for progression-free survival, overall survival, objective response rate, and decrement rate of grade 3-4 assessment. **Supplementary Table 2.** Comparisons of the fit of consistency and inconsistency models.

**Additional file 6: Supplementary Figure 3. **Bayesian ranking profile based on the SUCRA results of disease-controlled rate (DCR), decrement rate of any grade toxicity assessment, and rate of side effects leading to discontinuation and death.

**Additional file 7: Supplementary Figure 4.** Bayesian ranking profile based on the SUCRA results of decrement rate of toxicity assessment on seven commonly reported adverse events, including hematological (anemia, neutropenia, and thrombocytopenia) and non-hematological (nausea/vomiting, fatigue, diarrhea, and asthenia) adverse events.

**Additional file 8.**

## Data Availability

All data generated or analyzed during this study are included in this published article and its supplementary information files.

## Abbreviations

CI: Confidence interval
CTLA-4: Anti-cytotoxic T-lymphocyte antigen 4
DCR: Disease-controlled rate
HR: Hazard ratio
ICIs: Immune checkpoint inhibitors
NMA: Bayesian Network Meta-analysis
NSCLC: Non-small cell lung cancer
OS: Overall survival
ORR: Objective response rate
OR: Odds ratio
PD-1: Programmed death 1
PFS: Progression free survival
RCTs: Randomized controlled trials
SE: Standard error
SUCRA: Surface under the cumulative ranking
TKIs: Tyrosine kinase inhibitors
VEGF: Vascular endothelial growth factor
