# Supplementary material for: Role of antiangiogenic agents in first-line treatment for advanced NSCLC in the era of immunotherapy
Source: BMC Cancer. 2023 Jan 21;23:72. doi: 10.1186/s12885-022-10446-1 (PMC9862794; doi:10.1186/s12885-022-10446-1)
Supplement: Supplementary file 2 — Additional file 2. [file 12885_2022_10446_MOESM2_ESM.pdf]

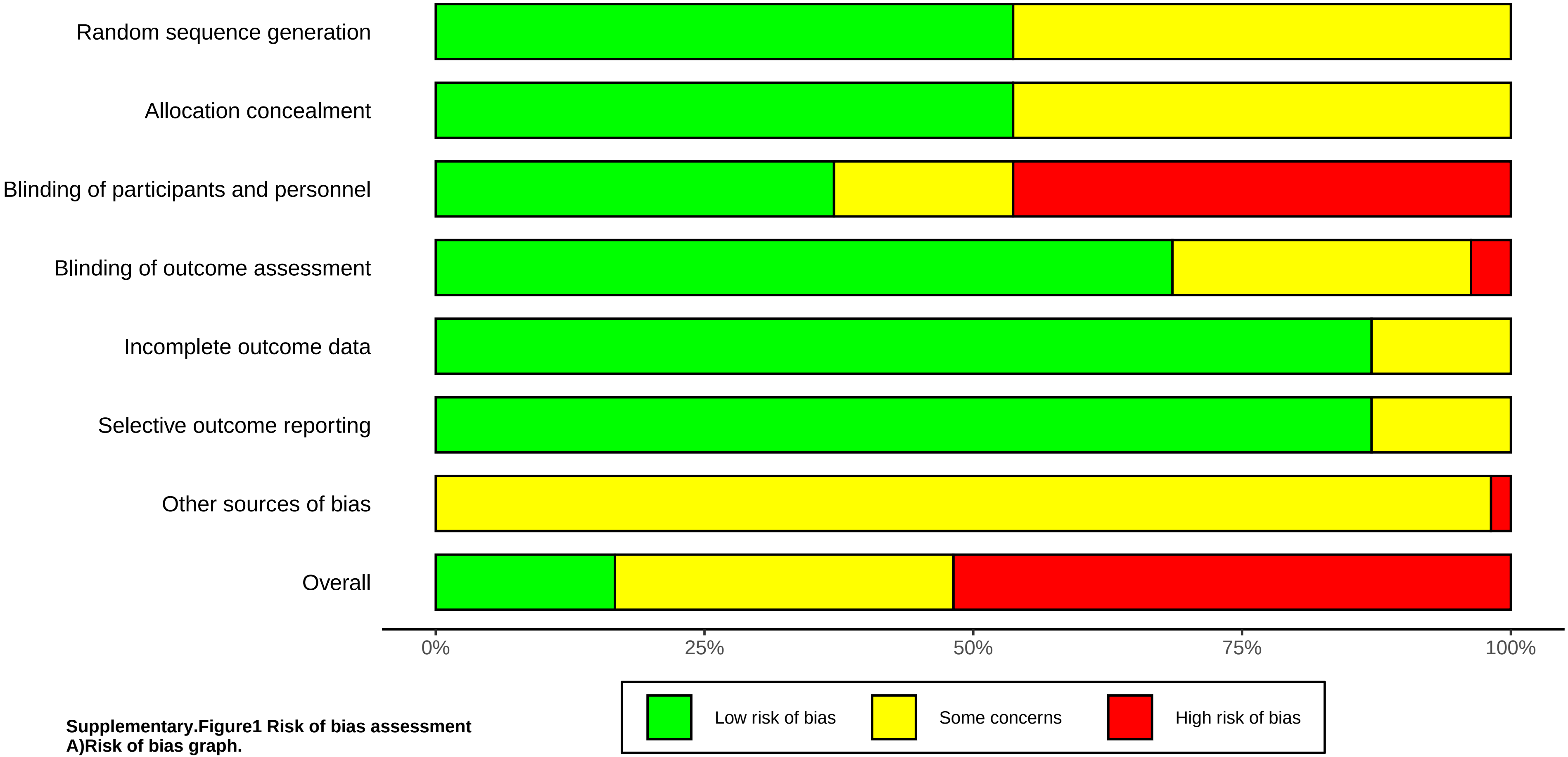

Supplementary.Figure1 Risk of bias assessment
A)Risk of bias graph.

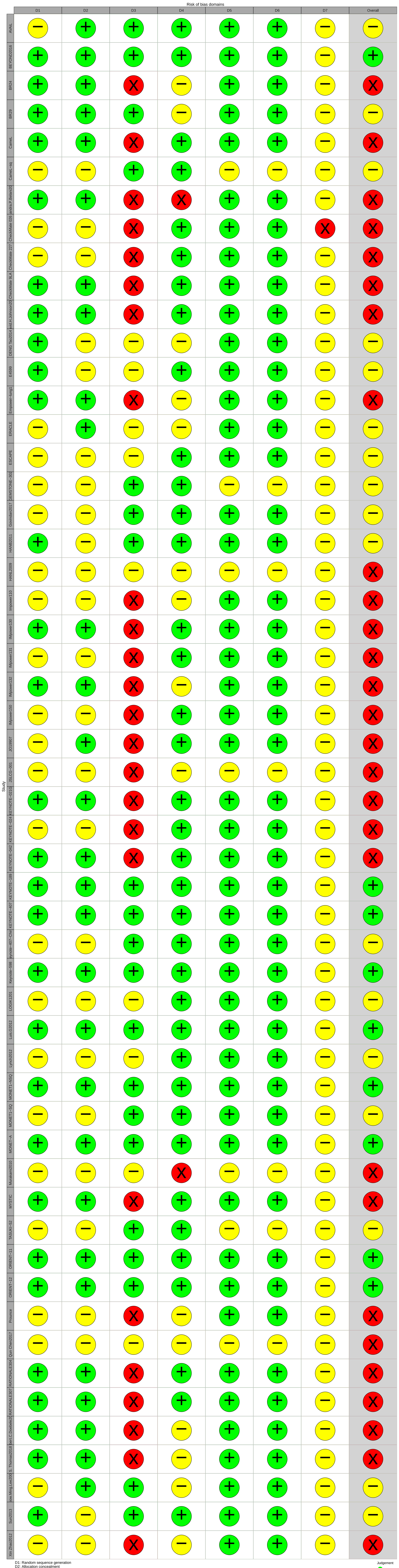

D1: Random sequence generation
D2: Allocation concealment
D3: Blinding of participants and personnel
D4: Blinding of outcome assessment
D5: Incomplete outcome data
D6: Selective outcome reporting
D7: Other sources of bias

Judgement
Low
Unclear
High

Supplementary.Figure1 Risk of bias assessment
B)Risk of bias summary
