# Supplementary material for: Role of antiangiogenic agents in first-line treatment for advanced NSCLC in the era of immunotherapy
Source: BMC Cancer. 2023 Jan 21;23:72. doi: 10.1186/s12885-022-10446-1 (PMC9862794; doi:10.1186/s12885-022-10446-1)

A.PFS in squamous NSCLC

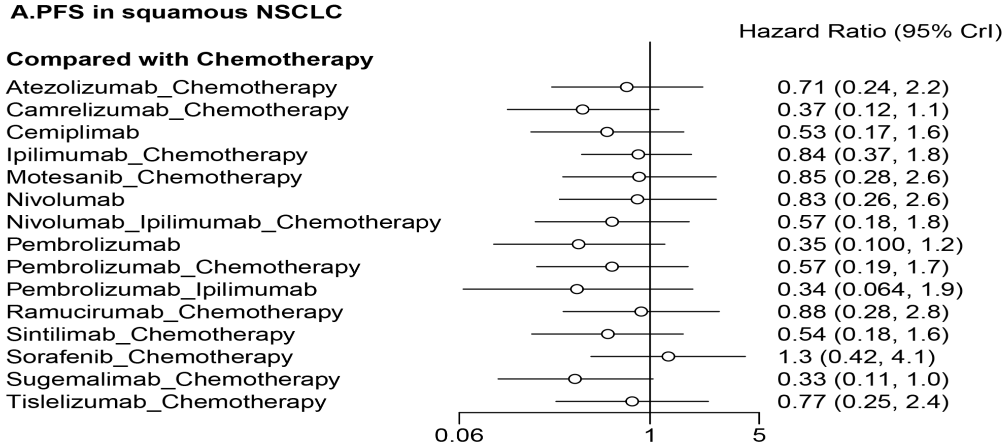

A. PFS in non-squamous NSCLC patients

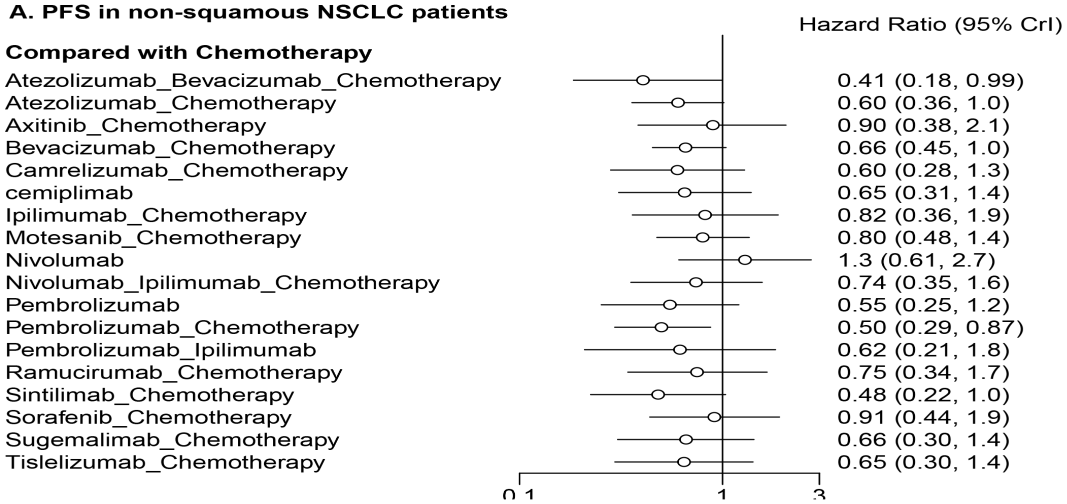

A. OS in squamous NSCLC patients

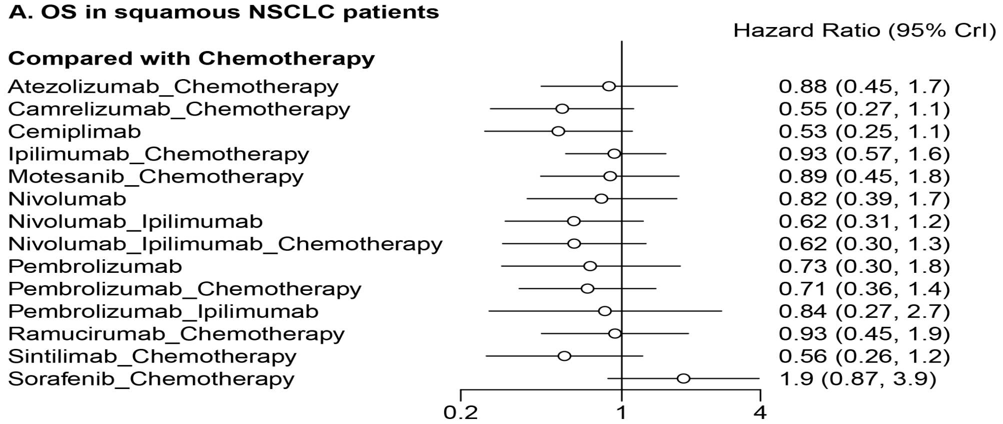

A.OS in non-squamous NSCLC patients

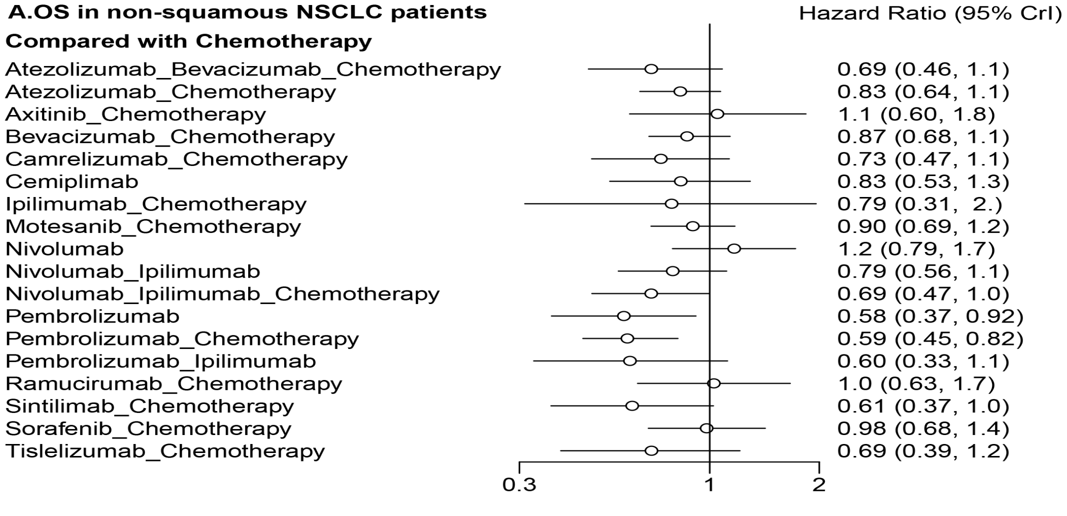

B: PFS in Male NSCLC patients

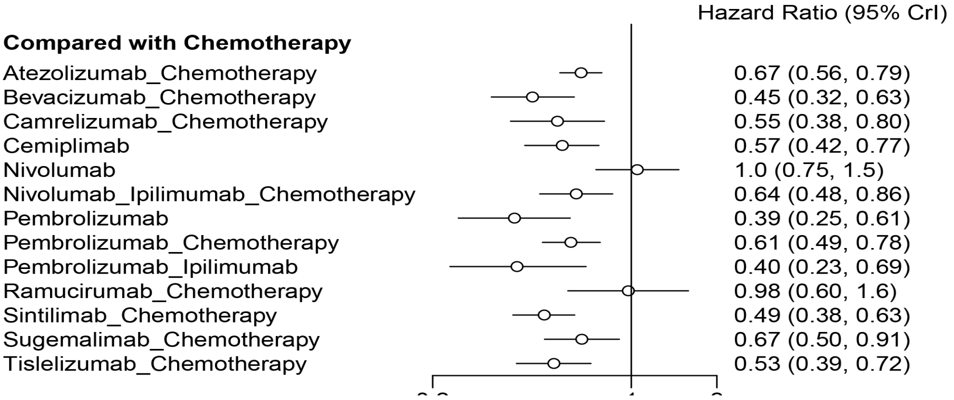

B: PFS in Female NSCLC patients

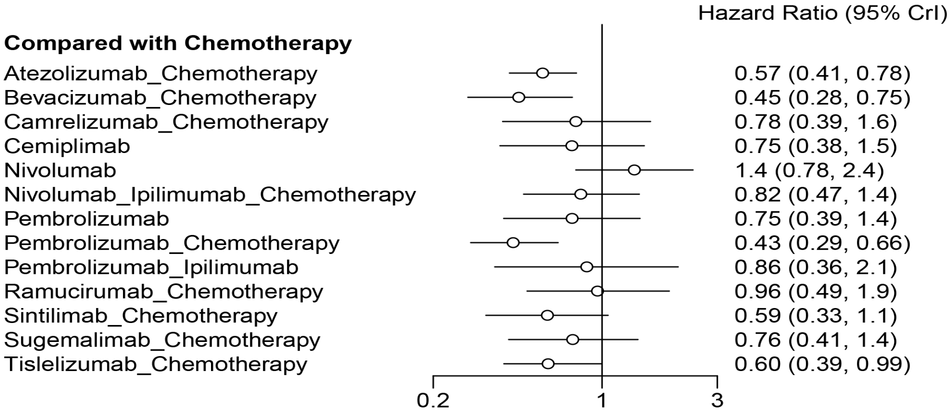

B: OS in Female NSCLC patients

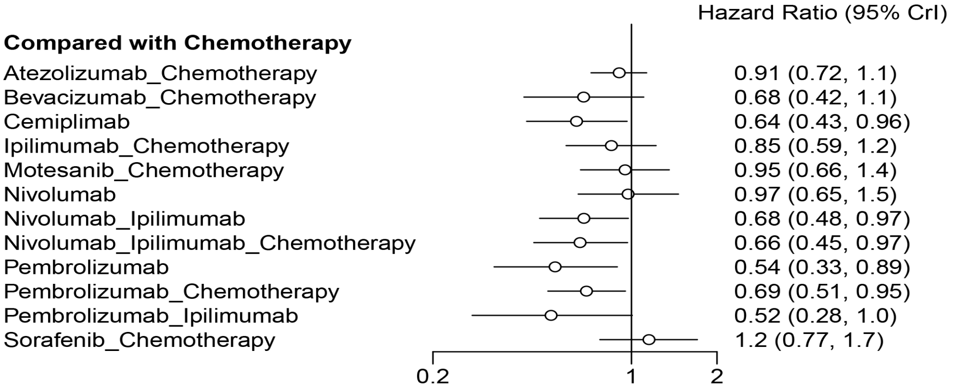

B: OS in Male NSCLC patients

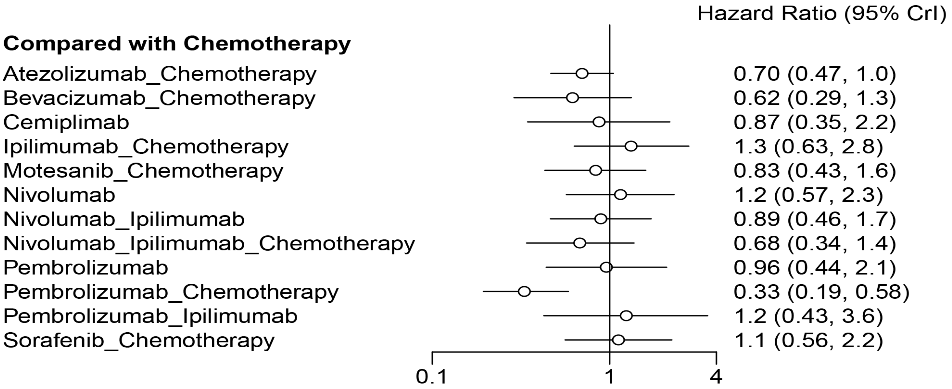

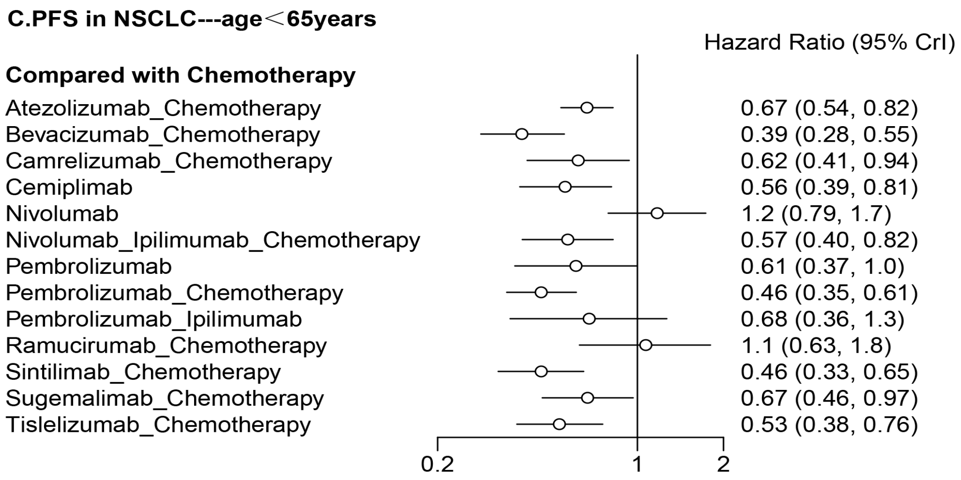
C.PFS in NSCLC---age＜65years
Hazard Ratio (95% CrI)
Compared with Chemotherapy
Atezolizumab_Chemotherapy 0.67 (0.54, 0.82)
Bevacizumab_Chemotherapy 0.39 (0.28, 0.55)
Camrelizumab_Chemotherapy 0.62 (0.41, 0.94)
Cemiplimab 0.56 (0.39, 0.81)
Nivolumab 1.2 (0.79, 1.7)
Nivolumab_Ipilimumab_Chemotherapy 0.57 (0.40, 0.82)
Pembrolizumab 0.61 (0.37, 1.0)
Pembrolizumab_Chemotherapy 0.46 (0.35, 0.61)
Pembrolizumab_Ipilimumab 0.68 (0.36, 1.3)
Ramucirumab_Chemotherapy 1.1 (0.63, 1.8)
Sintilimab_Chemotherapy 0.46 (0.33, 0.65)
Sugemalimab_Chemotherapy 0.67 (0.46, 0.97)
Tislelizumab_Chemotherapy 0.53 (0.38, 0.76)
0.2
1
2

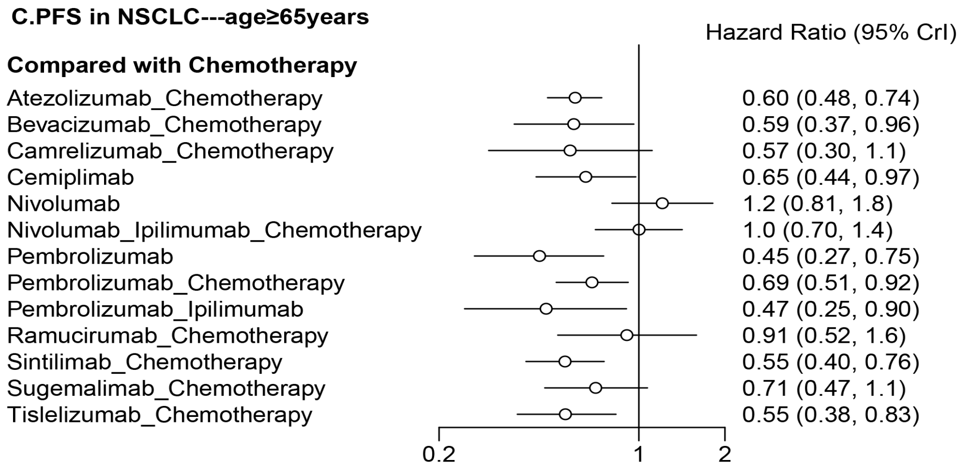
C.PFS in NSCLC---age≥65years
Hazard Ratio (95% CrI)
Compared with Chemotherapy
Atezolizumab_Chemotherapy 0.60 (0.48, 0.74)
Bevacizumab_Chemotherapy 0.59 (0.37, 0.96)
Camrelizumab_Chemotherapy 0.57 (0.30, 1.1)
Cemiplimab 0.65 (0.44, 0.97)
Nivolumab 1.2 (0.81, 1.8)
Nivolumab_Ipilimumab_Chemotherapy 1.0 (0.70, 1.4)
Pembrolizumab 0.45 (0.27, 0.75)
Pembrolizumab_Chemotherapy 0.69 (0.51, 0.92)
Pembrolizumab_Ipilimumab 0.47 (0.25, 0.90)
Ramucirumab_Chemotherapy 0.91 (0.52, 1.6)
Sintilimab_Chemotherapy 0.55 (0.40, 0.76)
Sugemalimab_Chemotherapy 0.71 (0.47, 1.1)
Tislelizumab_Chemotherapy 0.55 (0.38, 0.83)
0.2
1
2

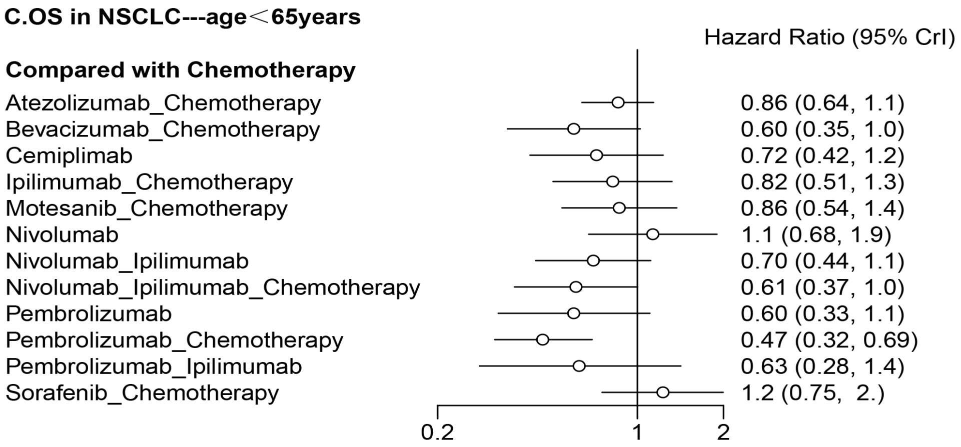
C.OS in NSCLC---age＜65years
Hazard Ratio (95% CrI)
Compared with Chemotherapy
Atezolizumab_Chemotherapy 0.86 (0.64, 1.1)
Bevacizumab_Chemotherapy 0.60 (0.35, 1.0)
Cemiplimab 0.72 (0.42, 1.2)
Ipilimumab_Chemotherapy 0.82 (0.51, 1.3)
Motesanib_Chemotherapy 0.86 (0.54, 1.4)
Nivolumab 1.1 (0.68, 1.9)
Nivolumab_Ipilimumab 0.70 (0.44, 1.1)
Nivolumab_Ipilimumab_Chemotherapy 0.61 (0.37, 1.0)
Pembrolizumab 0.60 (0.33, 1.1)
Pembrolizumab_Chemotherapy 0.47 (0.32, 0.69)
Pembrolizumab_Ipilimumab 0.63 (0.28, 1.4)
Sorafenib_Chemotherapy 1.2 (0.75, 2.)
0.2
1
2

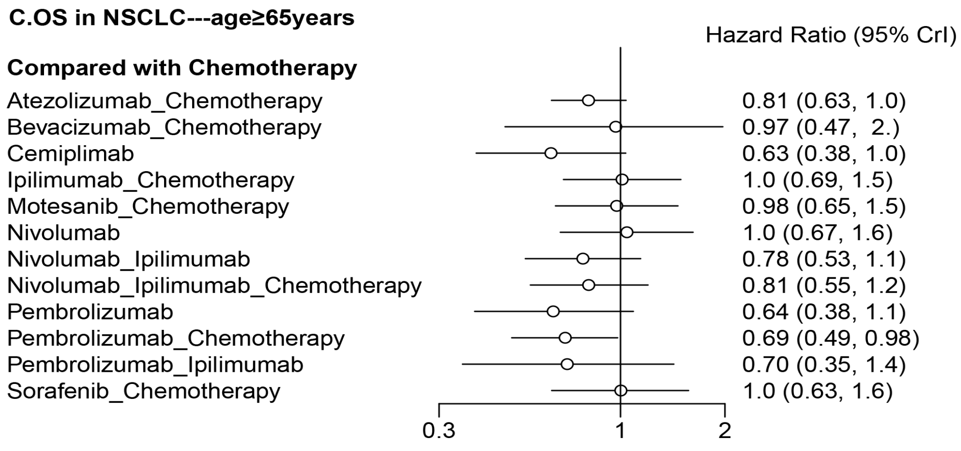
C.OS in NSCLC---age≥65years
Hazard Ratio (95% CrI)
Compared with Chemotherapy
Atezolizumab_Chemotherapy 0.81 (0.63, 1.0)
Bevacizumab_Chemotherapy 0.97 (0.47, 2.)
Cemiplimab 0.63 (0.38, 1.0)
Ipilimumab_Chemotherapy 1.0 (0.69, 1.5)
Motesanib_Chemotherapy 0.98 (0.65, 1.5)
Nivolumab 1.0 (0.67, 1.6)
Nivolumab_Ipilimumab 0.78 (0.53, 1.1)
Nivolumab_Ipilimumab_Chemotherapy 0.81 (0.55, 1.2)
Pembrolizumab 0.64 (0.38, 1.1)
Pembrolizumab_Chemotherapy 0.69 (0.49, 0.98)
Pembrolizumab_Ipilimumab 0.70 (0.35, 1.4)
Sorafenib_Chemotherapy 1.0 (0.63, 1.6)
0.3
1
2

D. PFS in Smokers---NSCLC patients

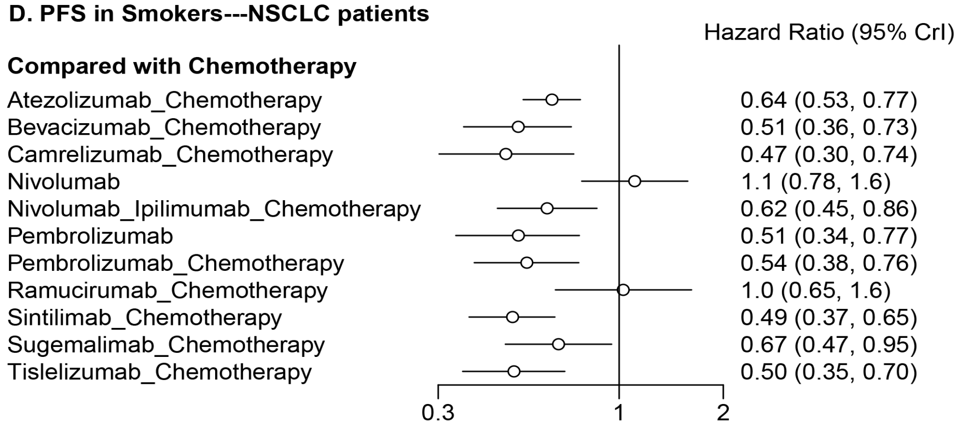

D. PFS in Non-smokers---NSCLC patients

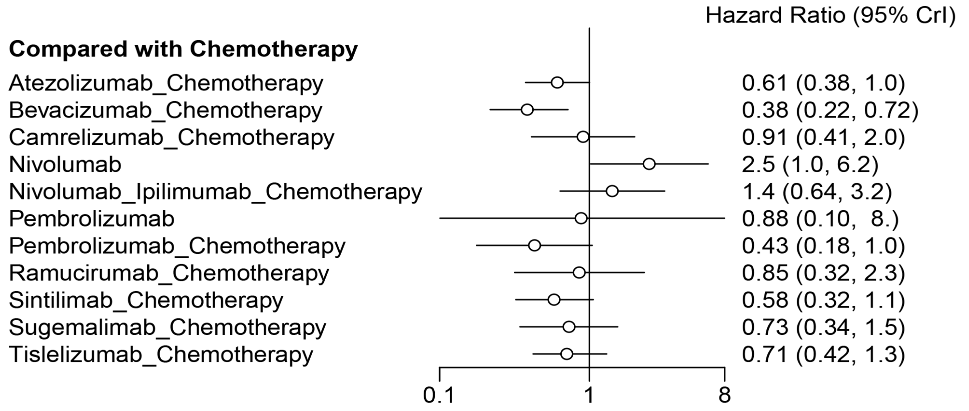

D. OS in Smokers---NSCLC patients

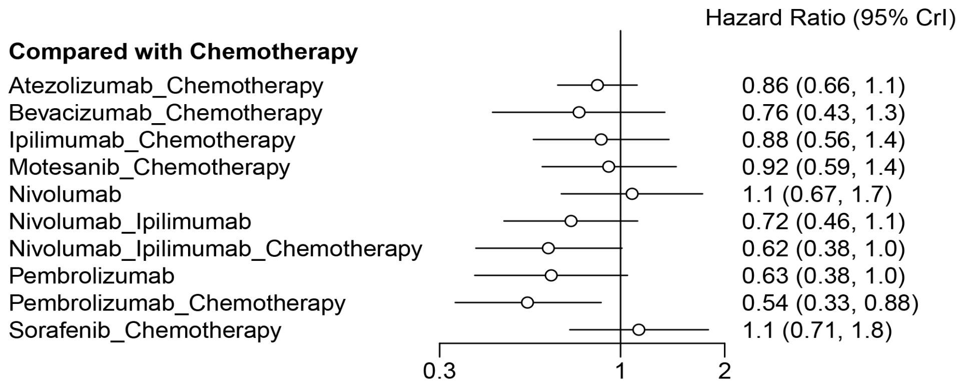

D: OS in Non-smokers---NSCLC patients

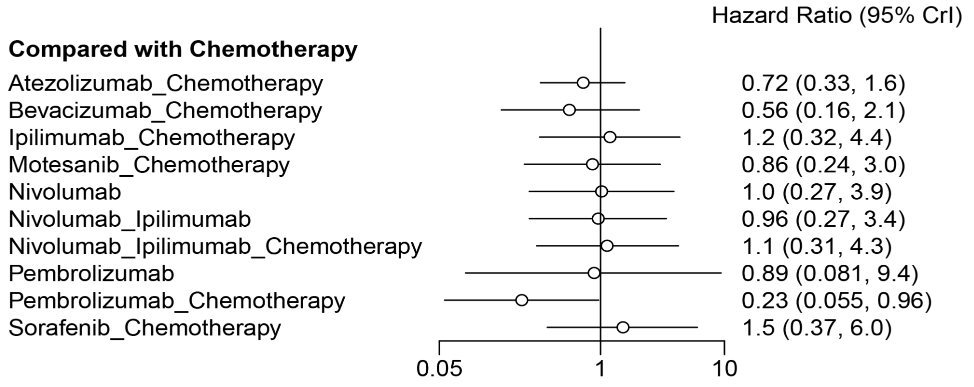

E. PFS in ECOG=0 NSCLC patients

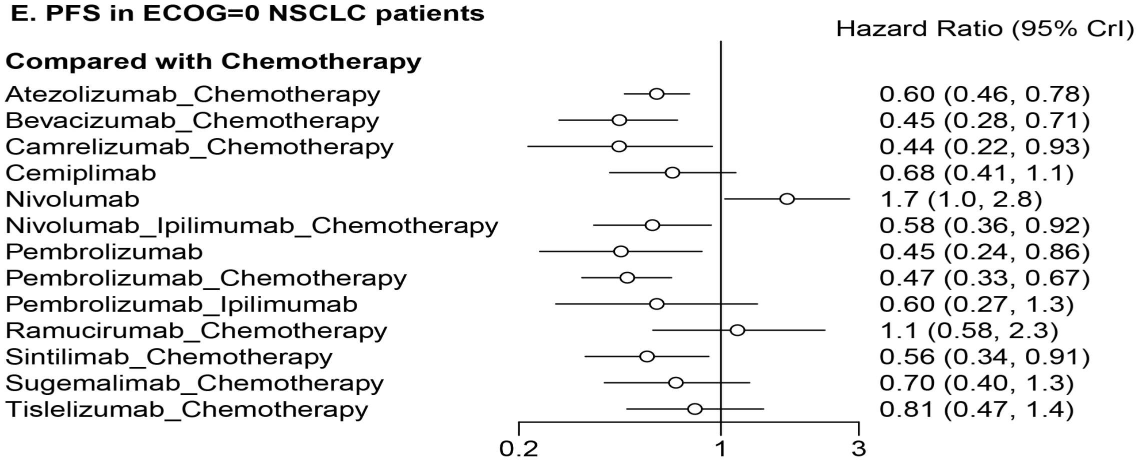

E. PFS in ECOG≥1 NSCLC patients

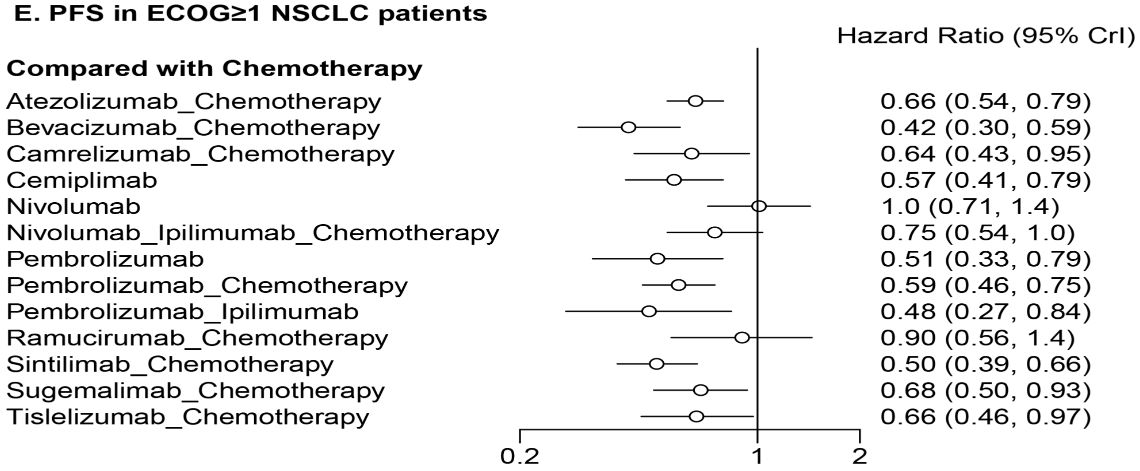

E. OS in ECOG=0 NSCLC patients

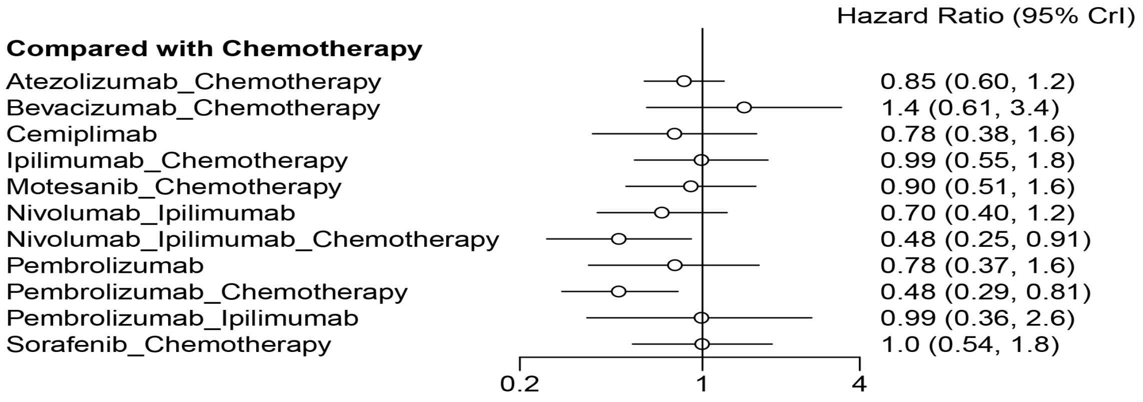

E. OS in ECOG≥1 NSCLC patients

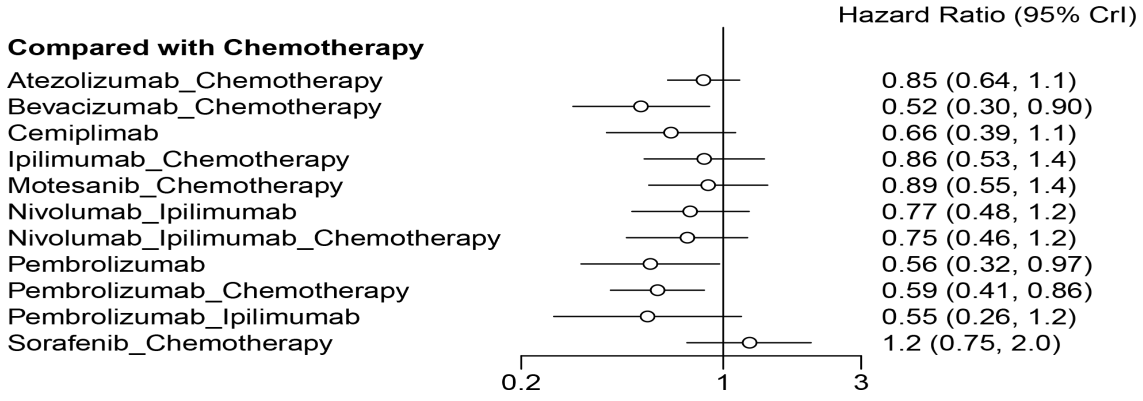

F.PFS in NSCLC patients with brain-metastasis

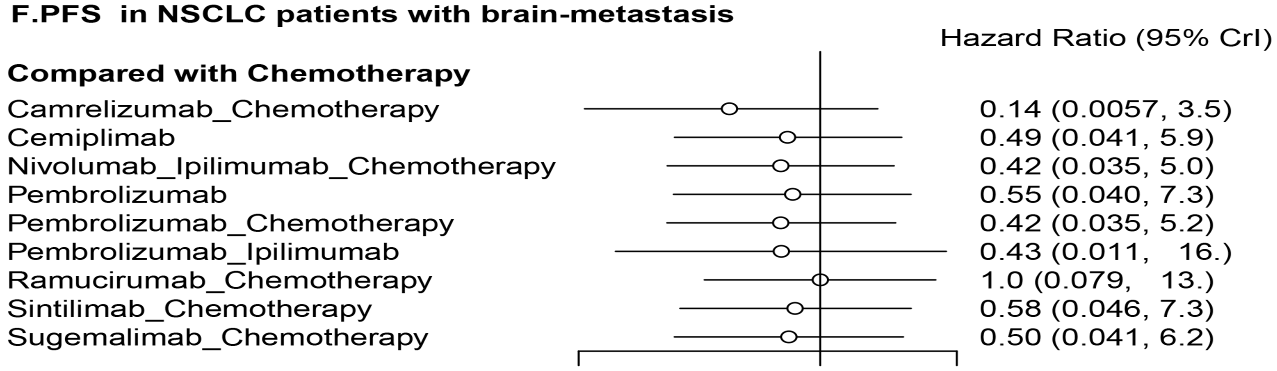

F.PFS in NSCLC patients without brain metastasis

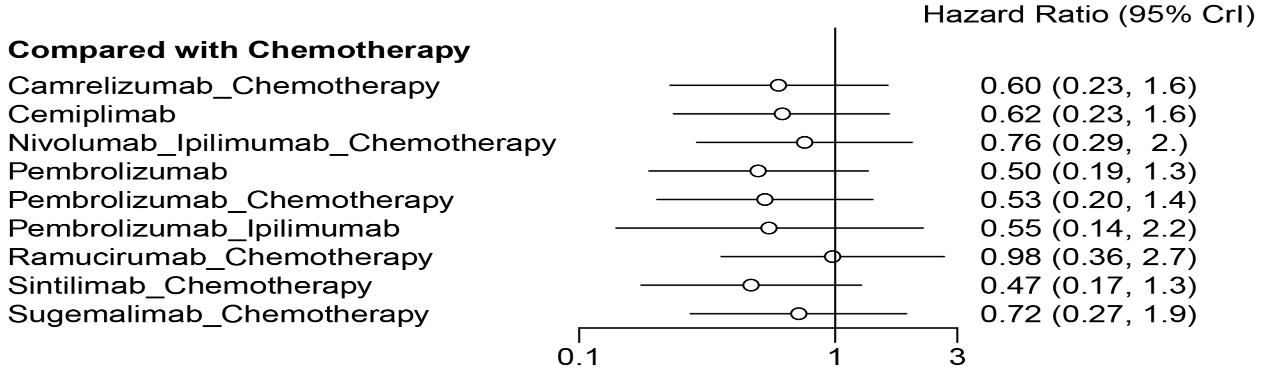

F.OS in NSCLC patients with brain-metastasis

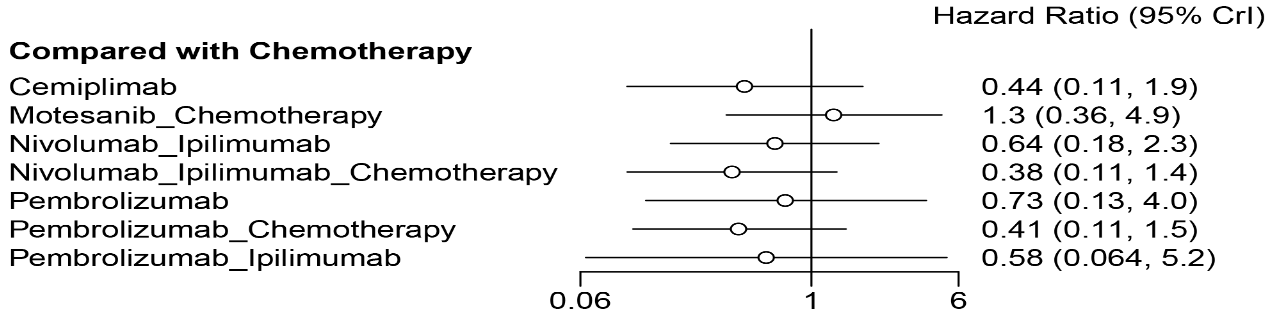

F.OS in NSCLC patients without brain-metastasis

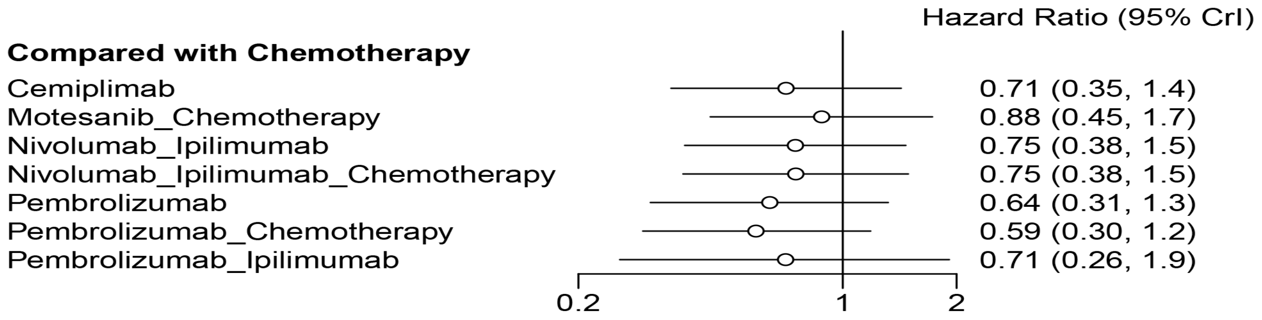

Supplement: Supplementary file 3 — Additional file 3: Supplementary Figure 1. Risk of bias assessment. A Risk of bias graph. B Risk of bias summary. [file 12885_2022_10446_MOESM3_ESM.pdf]
