# Supplementary material for: Role of antiangiogenic agents in first-line treatment for advanced NSCLC in the era of immunotherapy
Source: BMC Cancer. 2023 Jan 21;23:72. doi: 10.1186/s12885-022-10446-1 (PMC9862794; doi:10.1186/s12885-022-10446-1)
Supplement: Supplementary file 4 — Additional file 4: Supplementary Figure 2. Progression-free survival and overall survival comparison profile for advanced NSCLC under subgroup analysis stratified by histology, sex, age, smoking status, ECOG status, and brain metastasis or not. [file 12885_2022_10446_MOESM4_ESM.pdf]

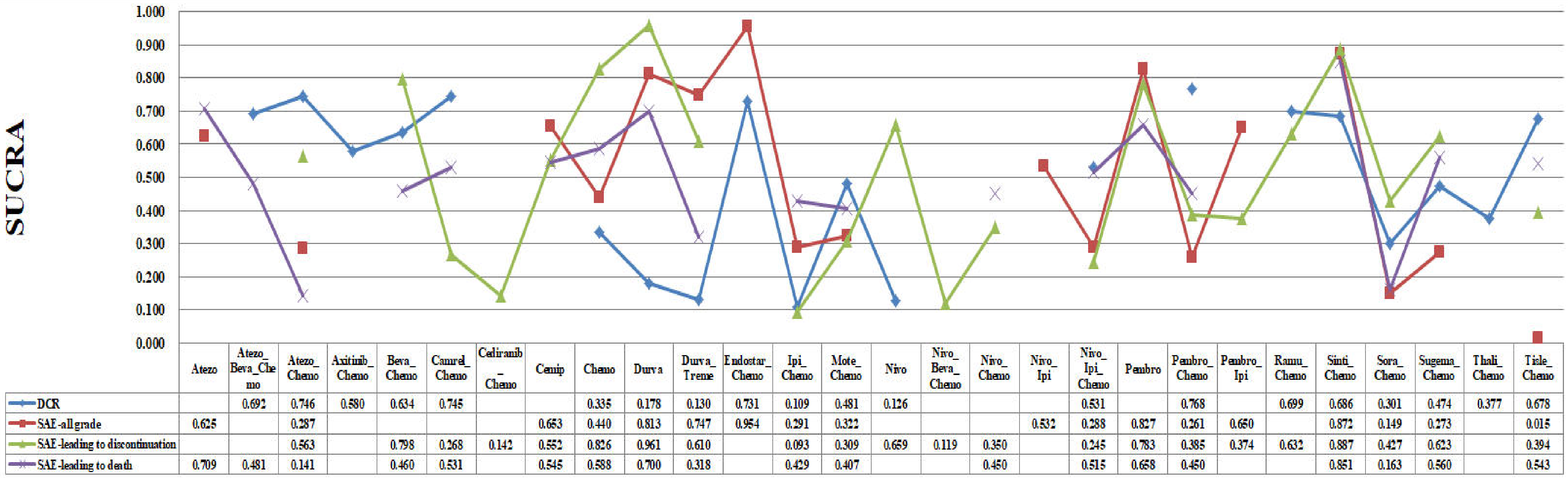

	Atezo	Atezo_Beva_Chemo	Atezo_Chemo	Axitinib_Chemo	Beva_Chemo	Camrel_Chemo	Cediranib_Chemo	Cemip	Chemo	Durva	Durva_Treme	Endostar_Chemo	Ipi_Chemo	Mote_Chemo	Nivo	Nivo_Beva_Chemo	Nivo_Chemo	Nivo_Ipi	Nivo_Ipi_Chemo	Pembro	Pembro_Chemo	Pembro_Ipi	Ramu_Chemo	Sinti_Chemo	Sora_Chemo	Sugema_Chemo	Thali_Chemo	Tisle_Chemo
DCR		0.692	0.746	0.580	0.634	0.745			0.335	0.178	0.130	0.731	0.109	0.481	0.126				0.531		0.768		0.699	0.686	0.301	0.474	0.377	0.678
SAE-all grade	0.625		0.287					0.653	0.440	0.813	0.747	0.954	0.291	0.322				0.532	0.288	0.827	0.261	0.650		0.872	0.149	0.273		0.015
SAE-leading to discontinuation			0.563		0.798	0.268	0.142	0.552	0.826	0.961	0.610		0.093	0.309	0.659	0.119	0.350		0.245	0.783	0.385	0.374	0.632	0.887	0.427	0.623		0.394
SAE-leading to death	0.709	0.481	0.141		0.460	0.531		0.545	0.588	0.700	0.318		0.429	0.407			0.450		0.515	0.658	0.450			0.851	0.163	0.560		0.543

Supplementary.Figure 3 Bayesian ranking profile based on the SUCRA results of disease controlled rate (DCR), decrement rate of any grade toxicity assessment and rate of side effects leading to discontinuation and death
