# Supplementary material for: Role of antiangiogenic agents in first-line treatment for advanced NSCLC in the era of immunotherapy
Source: BMC Cancer. 2023 Jan 21;23:72. doi: 10.1186/s12885-022-10446-1 (PMC9862794; doi:10.1186/s12885-022-10446-1)
Supplement: Supplementary file 5 — Additional file 5: Supplementary Table 1. Bayesian ranking results of network meta-analysis for progression-free survival, overall survival, objective response rate, and decrement rate of grade 3-4 assessment. Supplementary Table 2. Comparisons of the fit of consistency and inconsistency models. [file 12885_2022_10446_MOESM5_ESM.pdf]

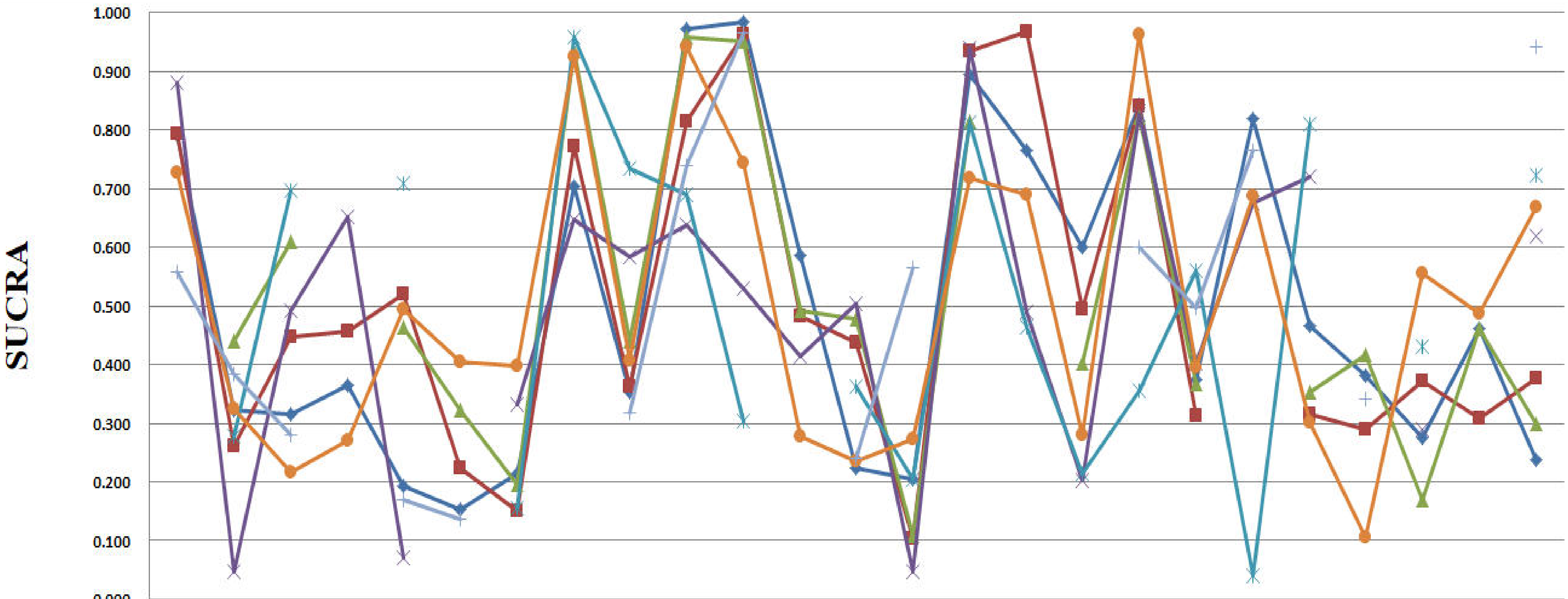

	Atezo	Atezo_ Beva_ Chemo	Atezo_ Chemo	Axitinib_ Chemo	Beva_ Chemo	Camrel_ Chemo	Cedir_ Chemo	Cemip	Chemo	Durva	Durva_ Treme	Endostar _ Chemo
anemia	0.791	0.323	0.314	0.364	0.194	0.153	0.214	0.704	0.353	0.973	0.983	0.587
neutropenia	0.793	0.262	0.447	0.456	0.520	0.222	0.150	0.772	0.363	0.814	0.963	0.482
thrombocytopenia		0.440	0.610		0.464	0.322	0.195	0.935	0.439	0.959	0.951	0.493
fatigue	0.880	0.047	0.491	0.652	0.070		0.332	0.647	0.584	0.638	0.531	0.413
diarrhea		0.278	0.697		0.709		0.154	0.958	0.734	0.689	0.303	
nausea/vomiting	0.728	0.326	0.218	0.270	0.494	0.405	0.398	0.926	0.407	0.942	0.743	0.279
asthenia	0.558	0.383	0.279		0.169	0.137			0.317	0.739	0.966	

	Ipi_ Chemo	Mote_ Chemo	Nivo	Nivo_ Chemo	Nivo_ Ipi_ Chemo	Pembro	Pembro_ Chemo	Pembro_ Ipi	Ramu_ Chemo	Sinti_ Chemo	Sora_ Chemo	Thali_ Chemo	Tisle_ Chemo
anemia	0.223	0.204	0.894	0.764	0.601	0.843	0.374	0.820	0.467	0.381	0.276	0.462	0.237
neutropenia	0.438	0.104	0.936	0.968	0.493	0.841	0.313		0.315	0.290	0.373	0.309	0.377
thrombocytopenia	0.478	0.108	0.815		0.403	0.821	0.367		0.353	0.416	0.169	0.462	0.299
fatigue	0.504	0.048	0.939	0.489	0.203	0.827	0.402	0.676	0.721		0.289		0.620
diarrhea	0.363	0.206	0.812	0.463	0.214	0.356	0.561	0.039	0.811		0.430		0.724
nausea/vomiting	0.236	0.273	0.719	0.691	0.279	0.964	0.396	0.688	0.301	0.106	0.555	0.488	0.669
asthenia	0.239	0.565				0.601	0.498	0.766		0.342			0.941

Supplementary.Figure 4 Bayesian ranking profile based on the SUCRA results of decrement rate of toxicity assessment on seven commonly reported adverse events, including hematological (anemia, neutropenia and thrombocytopenia) and non-hematological (nausea/vomiting, fatigue, diarrhea and asthenia) adverse events.
