# Supplementary material for: Role of antiangiogenic agents in first-line treatment for advanced NSCLC in the era of immunotherapy
Source: BMC Cancer. 2023 Jan 21;23:72. doi: 10.1186/s12885-022-10446-1 (PMC9862794; doi:10.1186/s12885-022-10446-1)
Supplement: Supplementary file 6 — Additional file 6: Supplementary Figure 3. Bayesian ranking profile based on the SUCRA results of disease-controlled rate (DCR), decrement rate of any grade toxicity assessment, and rate of side effects leading to discontinuation and death. [file 12885_2022_10446_MOESM6_ESM.docx]

**Supplementary.material2: Searching strategy**

Searching terms：

((“Carcinoma, Non-Small-Cell Lung” OR “NSCLC” OR “Non-Small Cell Lung Cancer”) OR (“Carcinoma” AND “Non-Small-Cell” AND “Lung”) OR "Non-Small-Cell Lung Carcinoma") AND ((“pembrolizumab”OR “lambrolizumab”OR “Keytruda”) OR (“atezolizumab” OR “Tecentriq”) OR(“Nivolumab” OR“OPDIVO”)OR (“Ipilimumab”OR“Yervoy”) OR (“Camrelizumab”) OR (“Tislelizumab”) OR (“Sintilimab”) OR (“Toripalimab”) OR (“Bevacizumab”OR“Avastin”) OR (“Endostar”OR“YH16”) OR (“Anlotinib”) OR (“Ramucirumab”) OR “Nintedanib” )AND (“trial” OR “study”).

Searching performed in the database of Web of Science was constrained to titles and abstracts while no limitations were applied in the PubMed, EMBASE and The Cochrane Library.
