# Supplementary material for: Role of antiangiogenic agents in first-line treatment for advanced NSCLC in the era of immunotherapy
Source: BMC Cancer. 2023 Jan 21;23:72. doi: 10.1186/s12885-022-10446-1 (PMC9862794; doi:10.1186/s12885-022-10446-1)
Supplement: Supplementary file 7 — Additional file 7: Supplementary Figure 4. Bayesian ranking profile based on the SUCRA results of decrement rate of toxicity assessment on seven commonly reported adverse events, including hematological (anemia, neutropenia, and thrombocytopenia) and non-hematological (nausea/vomiting, fatigue, diarrhea, and asthenia) adverse events. [file 12885_2022_10446_MOESM7_ESM.docx]

51. Kubota, K., et al., *Phase III, Randomized, Placebo-Controlled, Double-Blind Trial of Motesanib (AMG-706) in Combination With Paclitaxel and Carboplatin in East Asian Patients With Advanced Nonsquamous Non-Small-Cell Lung Cancer.* J Clin Oncol, 2017. **35**(32): p. 3662-3670.

52. Zinner, R.G., et al., *PRONOUNCE: randomized, open-label, phase III study of first-line pemetrexed + carboplatin followed by maintenance pemetrexed versus paclitaxel + carboplatin + bevacizumab followed by maintenance bevacizumab in patients ith advanced nonsquamous non-small-cell lung cancer.* J Thorac Oncol, 2015. **10**(1): p. 134-42.

53. Laurie, S.A., et al., *Randomised, double-blind trial of carboplatin and paclitaxel with daily oral cediranib or placebo in patients with advanced non-small cell lung cancer: NCIC Clinical Trials Group study BR29.* Eur J Cancer, 2014. **50**(4): p. 706-12.

54. Lee, S.M., et al., *Randomized double-blind placebo-controlled trial of thalidomide in combination with gemcitabine and Carboplatin in advanced non-small-cell lung cancer.* J Clin Oncol, 2009. **27**(31): p. 5248-54.

55. Niho, S., et al., *Randomized phase II study of first-line carboplatin-paclitaxel with or without bevacizumab in Japanese patients with advanced non-squamous non-small-cell lung cancer.* Lung Cancer, 2012. **76**(3): p. 362-7.

56. Fukuda, M., et al., *Randomized phase II study of pemetrexed or pemetrexed plus bevacizumab for elderly patients with previously untreated non-squamous non-small cell lung cancer: Results of the Lung Oncology Group in Kyushu (LOGIK1201).* Lung Cancer, 2019. **132**: p. 1-8.

57. Belani, C.P., et al., *Randomized phase II study of pemetrexed/cisplatin with or without axitinib for non-squamous non-small-cell lung cancer.* BMC Cancer, 2014. **14**: p. 290.

58. Zhao, X., et al., *A randomized phase II study of recombinant human endostatin plus gemcitabine/cisplatin compared with gemcitabine/cisplatin alone as first-line therapy in advanced non-small-cell lung cancer.* Invest New Drugs, 2012. **30**(3): p. 1144-9.

59. Johnson, D.H., et al., *Randomized phase II trial comparing bevacizumab plus carboplatin and paclitaxel with carboplatin and paclitaxel alone in previously untreated locally advanced or metastatic non-small-cell lung cancer.* J Clin Oncol, 2004. **22**(11): p. 2184-91.

60. Goss, G.D., et al., *Randomized, double-blind trial of carboplatin and paclitaxel with either daily oral cediranib or placebo in advanced non-small-cell lung cancer: NCIC clinical trials group BR24 study.* J Clin Oncol, 2010. **28**(1): p. 49-55.

61. Paz-Ares, L., et al., *A Randomized, Placebo-Controlled Trial of Pembrolizumab Plus Chemotherapy in Patients With Metastatic Squamous NSCLC: Protocol-Specified Final Analysis of KEYNOTE-407.* J Thorac Oncol, 2020. **15**(10): p. 1657-1669.

62. Zhou, C., et al., *Sintilimab Plus Platinum and Gemcitabine as First-Line Treatment for Advanced or Metastatic Squamous NSCLC: Results From a Randomized, Double-Blind, Phase 3 Trial (ORIENT-12).* J Thorac Oncol, 2021.

63. Chen, Q., et al., *A Study of Endostar Combined with Gemcitabine in the First-Line Treatment of the Elderly Patients with Advanced Non-Small Cell Lung Cancer.* Journal of Thoracic Oncology, 2017. **12**(1): p. S889-S890.

64. Wang, J., et al., *Tislelizumab Plus Chemotherapy vs Chemotherapy Alone as First-line Treatment for Advanced Squamous Non-Small-Cell Lung Cancer: A Phase 3 Randomized Clinical Trial.* JAMA Oncol, 2021. **7**(5): p. 709-717.

65. Li, X., W. Li, and L. Hou, *A Trial-Based Cost-Effectiveness Analysis of Bevacizumab and Chemotherapy Versus Chemotherapy Alone for Advanced Nonsquamous Non-Small-Cell Lung Cancer in China.* Value Health Reg Issues, 2019. **18**: p. 1-7.

66. Courtney, P.T., et al., *Cost-effectiveness of Nivolumab-Ipilimumab Combination Therapy for the Treatment of Advanced Non-Small Cell Lung Cancer.* JAMA Netw Open, 2021. **4**(5): p. e218787.

67. Goulart, B. and S. Ramsey, *A trial-based assessment of the cost-utility of bevacizumab and chemotherapy versus chemotherapy alone for advanced non-small cell lung cancer.* Value Health, 2011. **14**(6): p. 836-45.

68. Liu, G., et al., *Cost-Effectiveness Analysis of Atezolizumab Versus Chemotherapy as First-Line Treatment for Metastatic Non-Small-Cell Lung Cancer With Different PD-L1 Expression Status.* Front Oncol, 2021. **11**: p. 669195.

69. Peng, Y., et al., *First-Line Atezolizumab for Metastatic NSCLC with High PD-L1 Expression: A United States-Based Cost-Effectiveness Analysis.* Adv Ther, 2021. **38**(5): p. 2447-2457.

70. Lin, S., et al., *Cost-effectiveness of atezolizumab plus chemotherapy for advanced non-small-cell lung cancer.* Int J Clin Pharm, 2020. **42**(4): p. 1175-1183.

71. Yang, Z., et al., *First-line atezolizumab plus chemotherapy in advanced non-squamous non-small cell lung cancer: a cost-effectiveness analysis from China.* Expert Rev Pharmacoecon Outcomes Res, 2021: p. 1-7.

72. Page, R., et al., *Expanding indications of existing drugs and associated costs under risk reimbursement models.* Journal of Clinical Oncology, 2017. **35**: p. e18306-e18306.

73. Loong, H.H., et al., *Cost Effectiveness of PD-L1-Based Test-and-Treat Strategy with Pembrolizumab as the First-Line Treatment for Metastatic NSCLC in Hong Kong.* Pharmacoecon Open, 2020. **4**(2): p. 235-247.

74. Pinheiro, B., et al., *Cost-Effectiveness of Pembrolizumab for The First-Line Treatment of Metastatic Non-Small Cell Lung Carcinoma in Portugal.* Value in Health, 2017. **20**: p. A432.

75. Bhadhuri, A., et al., *Cost effectiveness of pembrolizumab vs chemotherapy as first-line treatment for metastatic NSCLC that expresses high levels of PD-L1 in Switzerland.* Swiss Med Wkly, 2019. **149**: p. w20170.

76. Huang, M., et al., *Cost Effectiveness of Pembrolizumab vs. Standard-of-Care Chemotherapy as First-Line Treatment for Metastatic NSCLC that Expresses High Levels of PD-L1 in the United States.* Pharmacoeconomics, 2017. **35**(8): p. 831-844.

77. Insinga, R.P., et al., *Cost-effectiveness of pembrolizumab in combination with chemotherapy versus chemotherapy and pembrolizumab monotherapy in the first-line treatment of squamous non-small-cell lung cancer in the US.* Curr Med Res Opin, 2019. **35**(7): p. 1241-1256.

78. Chouaid, C., et al., *COST-EFFECTIVENESS OF PEMBROLIZUMAB (KEYTRUDA (R)) IN COMBINATION WITH CHEMOTHERAPY FOR FIRST-LINE TREATMENT OF METASTATIC SQUAMOUS NON-SMALL CELL LUNG CANCER (NSCLC) IN FRANCE.* Value in Health, 2020. **23**: p. S433-S433.

79. Sezer, A., et al., *Cemiplimab monotherapy for first-line treatment of advanced non-small-cell lung cancer with PD-L1 of at least 50%: a multicentre, open-label, global, phase 3, randomised, controlled trial.* Lancet, 2021. **397**(10274): p. 592-604.

80. Zhou, C., et al., *GEMSTONE-302: A phase III study of platinum-based chemotherapy (chemo) with placebo or CS1001, an antiPDL1 antibody, for first-line (1L) advanced non-small cell lung cancer (NSCLC).* Annals of Oncology, 2020. **31**: p. S1386-S1386.

81. Zhou, C., et al., *96O Camrelizumab or placebo plus carboplatin and paclitaxel as first-line treatment for advanced squamous NSCLC (CameL-sq): A randomized, double-blind, multicenter, phase III trial.* Journal of Thoracic Oncology, 2021. **16**: p. S748.

82. Galetta, D., et al., *Cisplatin/Pemetrexed Followed by Maintenance Pemetrexed Versus Carboplatin/Paclitaxel/Bevacizumab Followed by Maintenance Bevacizumab in Advanced Nonsquamous Lung Cancer: The GOIM (Gruppo Oncologico Italia Meridionale) ERACLE Phase III Randomized Trial.* Clin Lung Cancer, 2015. **16**(4): p. 262-73.

83. Huang, J.A., et al., *Interim report on CIV recombinant human endostatin in combination with docetaxel/cisplatin (DP)) in comparison to DP in the first-line treatment of phase IIIb/IV squamous-cell NSCLC (JSLCG-001): A multicenter, open-label, randomized phase III controlled study (NCT: 02513342).* Journal of Clinical Oncology, 2019. **37**: p. e20541-e20541.

84. Han, L. and D. Xing, *Clinical observation of Endostar combined with TP chemotherapy in the treatment of advanced stage NSCLC.* 2009. **36**: p. 1205-1207.

85. Murakami, H., et al., *RANDOMIZED PHASE II STUDY OF BEVACIZUMAB COMBINED WITH CBDCA-PTX IN JAPANESE PATIENTS WITH ADVANCED NON-SQ NSCLC*. 2010. 11-12.

86. Wang, J., et al., *[Results of randomized, multicenter, double-blind phase III trial of rh-endostatin (YH-16) in treatment of advanced non-small cell lung cancer patients].* Zhongguo Fei Ai Za Zhi, 2005. **8**(4): p. 283-90.

87. Han, B.H., et al., *[A multicenter, randomized, double-blind, placebo-controlled safety study to evaluate the clinical effects and quality of life of paclitaxel-carboplatin (PC) alone or combined with endostar for advanced non-small cell lung cancer (NSCLC)].* Zhonghua Zhong Liu Za Zhi, 2011. **33**(11): p. 854-9.

88. Chen, J.H., et al., *Indirect comparison of efficacy and safety between immune checkpoint inhibitors and antiangiogenic therapy in advanced non-small-cell lung cancer.* Sci Rep, 2018. **8**(1): p. 9686.

89. Shiraishi, Y., et al., *Treatment Rationale and Design for APPLE (WJOG11218L): A Multicenter, Open-Label, Randomized Phase 3 Study of Atezolizumab and Platinum/Pemetrexed With or Without Bevacizumab for Patients With Advanced Nonsquamous Non-Small-Cell Lung Cancer.* Clin Lung Cancer, 2020. **21**(5): p. 472-476.

90. Taylor, M.H., et al., *The LEAP program: lenvatinib plus pembrolizumab for the treatment of advanced solid tumors.* Future Oncol, 2021. **17**(6): p. 637-648.

91. Ribas, A. and J.D. Wolchok, *Cancer immunotherapy using checkpoint blockade.* Science, 2018. **359**(6382): p. 1350-1355.

92. Zhang, B., et al., *Predictive effect of PD-L1 expression for immune checkpoint inhibitor (PD-1/PD-L1 inhibitors) treatment for non-small cell lung cancer: A meta-analysis.* Int Immunopharmacol, 2020. **80**: p. 106214.

93. Socinski, M.A., et al., *Current and Emergent Therapy Options for Advanced Squamous Cell Lung Cancer.* J Thorac Oncol, 2018. **13**(2): p. 165-183.

94. Hiraoka, K., et al., *Concurrent infiltration by CD8+ T cells and CD4+ T cells is a favourable prognostic factor in non-small-cell lung carcinoma.* Br J Cancer, 2006. **94**(2): p. 275-80.

95. Ruffini, E., et al., *Clinical significance of tumor-infiltrating lymphocytes in lung neoplasms.* Ann Thorac Surg, 2009. **87**(2): p. 365-71; discussion 371-2.

96. Ernani, V. and T.E. Stinchcombe, *Management of Brain Metastases in Non-Small-Cell Lung Cancer.* J Oncol Pract, 2019. **15**(11): p. 563-570.
