# Supplementary material for: Role of antiangiogenic agents in first-line treatment for advanced NSCLC in the era of immunotherapy
Source: BMC Cancer. 2023 Jan 21;23:72. doi: 10.1186/s12885-022-10446-1 (PMC9862794; doi:10.1186/s12885-022-10446-1)
Supplement: Supplementary file 8 — Additional file 8. [file 12885_2022_10446_MOESM8_ESM.docx]

**Supplementary.Table.1** Bayesian ranking results of network meta-analysis for progression free survival, overall survival, objective response rate and decrement rate of grade 3-4 assessment

|  | **11** | **2** | **3** | **4** | **5** | **6** | **7** | **8** | **9** | **10** | **11** | **12** | **13** | **14** | **15** | **16** | **17** | **18** | **19** | **20** | **21** | **22** | **23** | **24** | **25** | **26** |
| --- | --- | --- | --- | --- | --- | --- | --- | --- | --- | --- | --- | --- | --- | --- | --- | --- | --- | --- | --- | --- | --- | --- | --- | --- | --- | --- |
| **Progression free survival** | | | | | | | | | | | | | | | | | | | | | | | | | | |
| **Atezolizumab** | 0 | 1 | 1 | 1 | 2 | 2 | 3 | 4 | 5 | 5 | 6 | 6 | 6 | 6 | 6 | 6 | 6 | 6 | 5 | 5 | 4 | 4 | 3 | 3 | 3 | 1 |
| **Atezolizumab_Bevacizumab_Chemotherapy** | 21 | 19 | 13 | 10 | 8 | 7 | 5 | 4 | 3 | 2 | 2 | 1 | 1 | 1 | 1 | 0 | 0 | 0 | 0 | 0 | 0 | 0 | 0 | 0 | 0 | 0 |
| **Atezolizumab_Chemotherapy** | 0 | 0 | 1 | 2 | 4 | 6 | 9 | 11 | 13 | 13 | 11 | 9 | 7 | 5 | 4 | 3 | 2 | 1 | 1 | 0 | 0 | 0 | 0 | 0 | 0 | 0 |
| **Axitinib_Chemotherapy** | 1 | 1 | 1 | 1 | 1 | 2 | 2 | 3 | 3 | 3 | 3 | 4 | 4 | 4 | 4 | 4 | 5 | 5 | 5 | 5 | 5 | 5 | 6 | 7 | 8 | 9 |
| **Bevacizumab_Chemotherapy** | 0 | 0 | 0 | 0 | 0 | 0 | 1 | 2 | 4 | 7 | 10 | 12 | 13 | 13 | 11 | 9 | 7 | 5 | 3 | 2 | 1 | 0 | 0 | 0 | 0 | 0 |
| **Camrelizumab_Chemotherapy** | 13 | 17 | 16 | 14 | 11 | 9 | 7 | 5 | 3 | 2 | 1 | 1 | 1 | 0 | 0 | 0 | 0 | 0 | 0 | 0 | 0 | 0 | 0 | 0 | 0 | 0 |
| **Cediranib_Chemotherapy** | 0 | 0 | 0 | 0 | 0 | 1 | 1 | 2 | 2 | 3 | 4 | 5 | 5 | 6 | 7 | 8 | 8 | 8 | 8 | 7 | 6 | 5 | 5 | 4 | 3 | 1 |
| **Cemiplimab** | 3 | 5 | 6 | 7 | 7 | 8 | 9 | 8 | 8 | 7 | 6 | 5 | 4 | 3 | 3 | 2 | 2 | 2 | 1 | 1 | 1 | 1 | 1 | 0 | 0 | 0 |
| **Chemotherapy** | 0 | 0 | 0 | 0 | 0 | 0 | 0 | 0 | 0 | 0 | 0 | 0 | 0 | 0 | 0 | 0 | 1 | 3 | 7 | 14 | 22 | 24 | 18 | 8 | 2 | 0 |
| **Durvalumab** | 0 | 0 | 0 | 1 | 1 | 1 | 1 | 2 | 2 | 3 | 3 | 4 | 4 | 4 | 5 | 5 | 6 | 6 | 6 | 6 | 6 | 6 | 7 | 8 | 7 | 4 |
| **Durvalumab_Tremelimumab** | 0 | 0 | 0 | 0 | 0 | 0 | 0 | 0 | 1 | 1 | 1 | 1 | 1 | 2 | 2 | 3 | 3 | 4 | 4 | 5 | 5 | 6 | 9 | 13 | 17 | 20 |
| **Ipilimumab_Chemotherapy** | 0 | 0 | 0 | 0 | 0 | 0 | 0 | 0 | 1 | 1 | 1 | 2 | 3 | 4 | 4 | 6 | 7 | 8 | 9 | 9 | 9 | 9 | 9 | 9 | 7 | 3 |
| **Motesanib_Chemotherapy** | 0 | 0 | 0 | 0 | 0 | 0 | 1 | 1 | 2 | 3 | 4 | 6 | 7 | 8 | 10 | 10 | 10 | 10 | 8 | 6 | 5 | 3 | 2 | 2 | 1 | 0 |
| **Nivolumab** | 0 | 0 | 0 | 0 | 0 | 0 | 0 | 0 | 0 | 0 | 0 | 1 | 1 | 1 | 1 | 2 | 2 | 3 | 3 | 4 | 4 | 6 | 8 | 13 | 19 | 31 |
| **Nivolumab_Bevacizumab_Chemotheray** | 37 | 18 | 11 | 8 | 6 | 5 | 4 | 3 | 2 | 2 | 1 | 1 | 1 | 1 | 0 | 0 | 0 | 0 | 0 | 0 | 0 | 0 | 0 | 0 | 0 | 0 |
| **Nivolumab_Ipilimumab** | 2 | 3 | 4 | 5 | 6 | 7 | 8 | 8 | 8 | 7 | 7 | 6 | 5 | 4 | 4 | 3 | 3 | 2 | 2 | 1 | 1 | 1 | 1 | 1 | 0 | 0 |
| **Nivolumab_Ipilimumab_Chemotherapy** | 1 | 2 | 2 | 3 | 4 | 5 | 6 | 7 | 7 | 7 | 7 | 7 | 6 | 6 | 5 | 5 | 4 | 4 | 3 | 2 | 2 | 2 | 1 | 1 | 1 | 0 |
| **Pembrolizumab** | 0 | 0 | 0 | 0 | 1 | 1 | 2 | 2 | 3 | 5 | 6 | 7 | 7 | 8 | 9 | 9 | 8 | 8 | 7 | 5 | 4 | 3 | 2 | 2 | 1 | 0 |
| **Pembrolizumab_Chemotherapy** | 6 | 12 | 17 | 18 | 16 | 12 | 8 | 5 | 3 | 2 | 1 | 0 | 0 | 0 | 0 | 0 | 0 | 0 | 0 | 0 | 0 | 0 | 0 | 0 | 0 | 0 |
| **Pembrolizumab_Ipilimumab** | 1 | 1 | 1 | 1 | 2 | 2 | 2 | 3 | 3 | 4 | 4 | 4 | 4 | 5 | 5 | 5 | 5 | 5 | 5 | 5 | 5 | 5 | 5 | 6 | 6 | 5 |
| **Ramucirumab_Chemotherapy** | 0 | 0 | 0 | 1 | 1 | 1 | 2 | 3 | 3 | 4 | 5 | 6 | 6 | 7 | 7 | 7 | 7 | 7 | 6 | 6 | 5 | 4 | 4 | 4 | 3 | 1 |
| **Sintilimab_Chemotherapy** | 6 | 9 | 12 | 12 | 13 | 12 | 10 | 8 | 6 | 4 | 3 | 2 | 1 | 1 | 1 | 0 | 0 | 0 | 0 | 0 | 0 | 0 | 0 | 0 | 0 | 0 |
| **Sorafenib_Chemotherapy** | 0 | 0 | 0 | 0 | 0 | 0 | 0 | 1 | 1 | 1 | 2 | 3 | 3 | 4 | 6 | 7 | 8 | 9 | 10 | 9 | 9 | 8 | 7 | 6 | 4 | 2 |
| **Sugemalimab_Chemotherapy** | 8 | 9 | 9 | 9 | 9 | 8 | 8 | 7 | 6 | 5 | 4 | 3 | 3 | 2 | 2 | 2 | 1 | 1 | 1 | 1 | 1 | 0 | 0 | 0 | 0 | 0 |
| **Thalidomide_Chemotherapy** | 0 | 0 | 0 | 0 | 0 | 0 | 0 | 0 | 0 | 1 | 1 | 1 | 1 | 2 | 2 | 2 | 3 | 4 | 4 | 5 | 6 | 7 | 10 | 13 | 18 | 20 |
| **Tislelizumab_Chemotherapy** | 1 | 3 | 5 | 7 | 8 | 10 | 11 | 11 | 10 | 8 | 6 | 5 | 4 | 3 | 2 | 2 | 1 | 1 | 1 | 0 | 0 | 0 | 0 | 0 | 0 | 0 |

|  | **1** | **2** | **3** | **4** | **5** | **6** | **7** | **8** | **9** | **10** | **11** | **12** | **13** | **14** | **15** | **16** | **17** | **18** | **19** | **20** | **21** | **22** | **23** | **24** | **25** | **26** | **27** |
| --- | --- | --- | --- | --- | --- | --- | --- | --- | --- | --- | --- | --- | --- | --- | --- | --- | --- | --- | --- | --- | --- | --- | --- | --- | --- | --- | --- |
| **Overall survival** | | | | | | | | | | | | | | | | | | | | | | | | | | | |
| **Atezolizumab** | 0 | 0 | 0 | 1 | 1 | 2 | 2 | 3 | 4 | 5 | 6 | 6 | 7 | 8 | 8 | 8 | 8 | 7 | 6 | 5 | 4 | 3 | 2 | 2 | 1 | 0 | 0 |
| **Atezolizumab_Bevacizumab_Chemotherapy** | 0 | 0 | 1 | 2 | 3 | 4 | 6 | 7 | 8 | 9 | 9 | 9 | 9 | 8 | 7 | 5 | 4 | 3 | 2 | 1 | 1 | 0 | 0 | 0 | 0 | 0 | 0 |
| **Atezolizumab_Chemotherapy** | 0 | 0 | 0 | 0 | 0 | 0 | 1 | 1 | 3 | 5 | 8 | 11 | 14 | 15 | 15 | 11 | 7 | 4 | 2 | 1 | 0 | 0 | 0 | 0 | 0 | 0 | 0 |
| **Axitinib_Chemotherapy** | 0 | 1 | 1 | 1 | 1 | 1 | 1 | 1 | 1 | 2 | 2 | 2 | 2 | 2 | 3 | 3 | 3 | 3 | 4 | 4 | 4 | 5 | 6 | 8 | 11 | 29 | 1 |
| **Bevacizumab_Chemotherapy** | 0 | 0 | 0 | 0 | 0 | 0 | 0 | 0 | 0 | 0 | 0 | 1 | 1 | 3 | 6 | 10 | 15 | 18 | 19 | 14 | 8 | 3 | 1 | 0 | 0 | 0 | 0 |
| **Camrelizumab_Chemotherapy** | 11 | 14 | 15 | 13 | 11 | 9 | 7 | 6 | 4 | 3 | 2 | 2 | 1 | 1 | 1 | 0 | 0 | 0 | 0 | 0 | 0 | 0 | 0 | 0 | 0 | 0 | 0 |
| **Cediranib_Chemotherapy** | 0 | 0 | 0 | 0 | 1 | 1 | 2 | 3 | 3 | 4 | 5 | 6 | 7 | 7 | 8 | 8 | 8 | 8 | 7 | 6 | 5 | 4 | 3 | 2 | 1 | 0 | 0 |
| **Cemiplimab** | 6 | 8 | 9 | 9 | 9 | 9 | 9 | 7 | 7 | 5 | 5 | 4 | 3 | 2 | 2 | 2 | 1 | 1 | 1 | 0 | 0 | 0 | 0 | 0 | 0 | 0 | 0 |
| **Chemotherapy** | 0 | 0 | 0 | 0 | 0 | 0 | 0 | 0 | 0 | 0 | 0 | 0 | 0 | 0 | 0 | 0 | 0 | 1 | 4 | 12 | 25 | 30 | 19 | 6 | 1 | 0 | 0 |
| **Durvalumab** | 4 | 6 | 7 | 8 | 8 | 8 | 8 | 8 | 7 | 6 | 5 | 4 | 4 | 3 | 2 | 2 | 2 | 1 | 1 | 1 | 0 | 0 | 0 | 0 | 0 | 0 | 0 |
| **Durvalumab_Tremelimumab** | 1 | 3 | 3 | 4 | 5 | 6 | 7 | 7 | 7 | 7 | 7 | 6 | 6 | 5 | 5 | 4 | 4 | 3 | 3 | 2 | 1 | 1 | 1 | 1 | 0 | 0 | 0 |
| **Endostar_Chemotherapy** | 0 | 0 | 0 | 0 | 0 | 0 | 0 | 0 | 0 | 0 | 0 | 0 | 0 | 0 | 0 | 0 | 0 | 0 | 0 | 0 | 0 | 0 | 0 | 0 | 0 | 1 | **99** |
| **Ipilimumab_Chemotherapy** | 0 | 0 | 0 | 0 | 0 | 0 | 0 | 0 | 0 | 0 | 1 | 1 | 2 | 3 | 4 | 6 | 8 | 10 | 12 | 13 | 12 | 9 | 8 | 6 | 3 | 1 | 0 |
| **Motesanib_Chemotherapy** | 0 | 0 | 0 | 0 | 0 | 0 | 0 | 0 | 1 | 1 | 2 | 3 | 5 | 7 | 9 | 12 | 13 | 14 | 12 | 9 | 6 | 3 | 2 | 1 | 0 | 0 | 0 |
| **Nivolumab** | 0 | 0 | 0 | 0 | 0 | 0 | 0 | 0 | 0 | 0 | 0 | 0 | 1 | 1 | 1 | 1 | 2 | 3 | 3 | 5 | 6 | 7 | 11 | 16 | 20 | 21 | 0 |
| **Nivolumab_Bevacizumab_Chemotherapy** | 2 | 3 | 3 | 4 | 4 | 5 | 5 | 6 | 6 | 6 | 6 | 6 | 6 | 5 | 5 | 5 | 4 | 4 | 3 | 3 | 2 | 2 | 2 | 2 | 1 | 1 | 0 |
| **Nivolumab_Ipilimumab** | 0 | 1 | 2 | 4 | 6 | 8 | 10 | 11 | 11 | 11 | 9 | 7 | 6 | 5 | 3 | 2 | 1 | 1 | 1 | 0 | 0 | 0 | 0 | 0 | 0 | 0 | 0 |
| **Nivolumab_Ipilimumab_Chemotherapy** | 5 | 10 | 11 | 12 | 12 | 11 | 9 | 7 | 6 | 4 | 3 | 3 | 2 | 1 | 1 | 1 | 0 | 0 | 0 | 0 | 0 | 0 | 0 | 0 | 0 | 0 | 0 |
| **Pembrolizumab** | 0 | 0 | 1 | 1 | 2 | 5 | 6 | 9 | 11 | 12 | 13 | 12 | 9 | 7 | 5 | 3 | 2 | 1 | 1 | 0 | 0 | 0 | 0 | 0 | 0 | 0 | 0 |
| **Pembrolizumab_Chemotherapy** | 7 | 14 | 17 | 18 | 15 | 11 | 7 | 5 | 3 | 2 | 1 | 0 | 0 | 0 | 0 | 0 | 0 | 0 | 0 | 0 | 0 | 0 | 0 | 0 | 0 | 0 | 0 |
| **Pembrolizumab_Ipilimumab** | 1 | 1 | 2 | 2 | 2 | 3 | 4 | 4 | 5 | 5 | 6 | 6 | 6 | 6 | 6 | 6 | 5 | 5 | 5 | 4 | 4 | 3 | 3 | 3 | 2 | 1 | 0 |
| **Ramucirumab_Chemotherapy** | 0 | 0 | 0 | 0 | 0 | 0 | 1 | 1 | 1 | 1 | 2 | 2 | 3 | 3 | 4 | 4 | 5 | 6 | 7 | 7 | 7 | 8 | 10 | 11 | 10 | 7 | 0 |
| **Sintilimab_Chemotherapy** | **30** | 17 | 12 | 9 | 7 | 6 | 4 | 4 | 3 | 2 | 2 | 1 | 1 | 1 | 1 | 0 | 0 | 0 | 0 | 0 | 0 | 0 | 0 | 0 | 0 | 0 | 0 |
| **Sorafenib_Chemotherapy** | 0 | 0 | 0 | 0 | 0 | 0 | 0 | 0 | 0 | 0 | 0 | 0 | 0 | 0 | 0 | 1 | 1 | 3 | 4 | 5 | 8 | 11 | 18 | 22 | 18 | 8 | 0 |
| **Sugemalimab_Chemotherapy** | 16 | 11 | 9 | 7 | 7 | 6 | 5 | 5 | 5 | 4 | 4 | 3 | 3 | 3 | 2 | 2 | 2 | 1 | 1 | 1 | 1 | 1 | 1 | 0 | 0 | 0 | 0 |
| **Thalidomide_Chemotherapy** | 0 | 0 | 0 | 0 | 0 | 0 | 0 | 0 | 0 | 0 | 0 | 0 | 0 | 0 | 0 | 0 | 1 | 1 | 2 | 3 | 4 | 6 | 10 | 18 | 28 | 28 | 0 |
| **Tislelizumab_Chemotherapy** | 16 | 10 | 7 | 6 | 5 | 5 | 5 | 4 | 4 | 4 | 4 | 3 | 3 | 3 | 3 | 2 | 2 | 2 | 2 | 2 | 1 | 1 | 1 | 2 | 2 | 1 | 0 |

| **Rate of grade3-4 toxicity assessment** | | | | | | | | | | | | | | | | | | | | | | | | | |
| --- | --- | --- | --- | --- | --- | --- | --- | --- | --- | --- | --- | --- | --- | --- | --- | --- | --- | --- | --- | --- | --- | --- | --- | --- | --- |
| **Atezolizumab** | 4 | 17 | 21 | 26 | 17 | 9 | 4 | 2 | 1 | 0 | 0 | 0 | 0 | 0 | 0 | 0 | 0 | 0 | 0 | 0 | 0 | 0 | 0 | 0 | 0 |
| **Atezolizumab_Bevacizumab_Chemotherapy** | 0 | 0 | 0 | 0 | 0 | 0 | 0 | 0 | 1 | 1 | 1 | 1 | 2 | 2 | 3 | 4 | 5 | 6 | 7 | 8 | 11 | 13 | 14 | 13 | 8 |
| **Atezolizumab_Chemotherapy** | 0 | 0 | 0 | 0 | 0 | 0 | 0 | 0 | 0 | 0 | 0 | 1 | 2 | 4 | 6 | 8 | 11 | 14 | 15 | 15 | 12 | 7 | 3 | 1 | 0 |
| **Bevacizumab_Chemotherapy** | 0 | 0 | 0 | 0 | 0 | 0 | 0 | 1 | 2 | 3 | 5 | 7 | 10 | 12 | 13 | 12 | 11 | 9 | 7 | 5 | 3 | 1 | 0 | 0 | 0 |
| **Camrelizumab_Chemotherapy** | 0 | 0 | 0 | 0 | 1 | 1 | 3 | 5 | 9 | 9 | 8 | 8 | 8 | 7 | 7 | 6 | 6 | 5 | 4 | 4 | 3 | 3 | 2 | 1 | 0 |
| **Cemiplimab** | 0 | 1 | 2 | 4 | 11 | 19 | 23 | 19 | 10 | 5 | 2 | 1 | 1 | 1 | 0 | 0 | 0 | 0 | 0 | 0 | 0 | 0 | 0 | 0 | 0 |
| **Chemotherapy** | 0 | 0 | 0 | 0 | 0 | 0 | 0 | 0 | 4 | 14 | 25 | 26 | 17 | 8 | 3 | 1 | 0 | 0 | 0 | 0 | 0 | 0 | 0 | 0 | 0 |
| **Durvalumab** | 8 | 31 | 24 | 20 | 10 | 4 | 1 | 1 | 0 | 0 | 0 | 0 | 0 | 0 | 0 | 0 | 0 | 0 | 0 | 0 | 0 | 0 | 0 | 0 | 0 |
| **Durvalumab_Tremelimumab** | 0 | 1 | 3 | 7 | 17 | 23 | 21 | 15 | 7 | 3 | 2 | 1 | 1 | 0 | 0 | 0 | 0 | 0 | 0 | 0 | 0 | 0 | 0 | 0 | 0 |
| **Endostar_Chemotherapy** | 2 | 6 | 7 | 9 | 13 | 13 | 12 | 11 | 7 | 4 | 3 | 2 | 2 | 2 | 1 | 1 | 1 | 1 | 1 | 0 | 0 | 0 | 0 | 0 | 0 |
| **Ipilimumab_Chemotherapy** | 0 | 0 | 0 | 0 | 0 | 0 | 0 | 0 | 0 | 0 | 0 | 1 | 1 | 2 | 3 | 4 | 5 | 6 | 8 | 11 | 14 | 17 | 16 | 9 | 2 |
| **Motesanib_Chemotherapy** | 0 | 0 | 0 | 0 | 0 | 0 | 0 | 0 | 0 | 0 | 0 | 0 | 0 | 0 | 0 | 0 | 0 | 1 | 2 | 3 | 7 | 14 | 25 | 36 | 11 |
| **Nivolumab** | 81 | 12 | 4 | 2 | 1 | 0 | 0 | 0 | 0 | 0 | 0 | 0 | 0 | 0 | 0 | 0 | 0 | 0 | 0 | 0 | 0 | 0 | 0 | 0 | 0 |
| **Nivolumab_Bevacizumab_Chemotherapy** | 0 | 0 | 0 | 0 | 1 | 1 | 2 | 4 | 6 | 7 | 7 | 6 | 7 | 7 | 7 | 7 | 6 | 6 | 6 | 5 | 5 | 4 | 3 | 2 | 1 |
| **Nivolumab_Ipilimumab** | 0 | 0 | 0 | 0 | 1 | 3 | 7 | 13 | 20 | 16 | 10 | 7 | 5 | 4 | 3 | 2 | 2 | 1 | 1 | 1 | 0 | 0 | 0 | 0 | 0 |
| **Nivolumab_Ipilimumab_Chemotherapy** | 0 | 0 | 0 | 0 | 0 | 0 | 0 | 1 | 1 | 2 | 2 | 3 | 4 | 6 | 6 | 7 | 8 | 9 | 10 | 10 | 10 | 8 | 6 | 4 | 1 |
| **Pembrolizumab** | 4 | 31 | 34 | 21 | 7 | 2 | 1 | 0 | 0 | 0 | 0 | 0 | 0 | 0 | 0 | 0 | 0 | 0 | 0 | 0 | 0 | 0 | 0 | 0 | 0 |
| **Pembrolizumab_Chemotherapy** | 0 | 0 | 0 | 0 | 0 | 0 | 0 | 0 | 1 | 2 | 3 | 5 | 8 | 10 | 12 | 12 | 12 | 11 | 9 | 7 | 4 | 2 | 1 | 0 | 0 |
| **Pembrolizumab_Ipilimumab** | 0 | 2 | 5 | 10 | 19 | 19 | 17 | 12 | 6 | 3 | 2 | 1 | 1 | 1 | 1 | 0 | 0 | 0 | 0 | 0 | 0 | 0 | 0 | 0 | 0 |
| **Ramucirumab_Chemotherapy** | 0 | 0 | 0 | 1 | 1 | 2 | 3 | 5 | 6 | 6 | 5 | 4 | 4 | 5 | 5 | 5 | 5 | 5 | 5 | 5 | 6 | 6 | 6 | 6 | 5 |
| **Sintilimab_Chemotherapy** | 0 | 0 | 0 | 0 | 0 | 0 | 1 | 2 | 4 | 5 | 6 | 8 | 9 | 10 | 10 | 9 | 8 | 7 | 6 | 5 | 4 | 3 | 1 | 1 | 0 |
| **Sorafenib_Chemotherapy** | 0 | 0 | 0 | 0 | 0 | 0 | 0 | 0 | 0 | 0 | 0 | 0 | 0 | 0 | 0 | 0 | 0 | 0 | 0 | 1 | 1 | 3 | 7 | 20 | 67 |
| **Sugemalimab_Chemotherapy** | 0 | 0 | 0 | 0 | 1 | 2 | 4 | 7 | 11 | 12 | 10 | 8 | 8 | 7 | 6 | 5 | 4 | 4 | 3 | 2 | 2 | 1 | 1 | 0 | 0 |
| **Thalidomide_Chemotherapy** | 0 | 0 | 0 | 0 | 0 | 0 | 1 | 2 | 3 | 5 | 5 | 6 | 8 | 9 | 9 | 9 | 9 | 8 | 7 | 6 | 5 | 4 | 2 | 1 | 0 |
| **Tislelizumab_Chemotherapy** | 0 | 0 | 0 | 0 | 0 | 0 | 0 | 0 | 0 | 1 | 1 | 2 | 2 | 3 | 4 | 5 | 7 | 8 | 10 | 11 | 13 | 13 | 10 | 6 | 2 |

| **Objective response rate** | | | | | | | | | | | | | | | | | | | | | | | | | | | |
| --- | --- | --- | --- | --- | --- | --- | --- | --- | --- | --- | --- | --- | --- | --- | --- | --- | --- | --- | --- | --- | --- | --- | --- | --- | --- | --- | --- |
| **Atezolizumab** | 0 | 0 | 0 | 0 | 0 | 0 | 0 | 0 | 0 | 0 | 0 | 0 | 1 | 1 | 1 | 1 | 2 | 3 | 5 | 6 | 7 | 7 | 9 | 12 | 14 | 16 | 14 |
| **Atezolizumab_Bevacizumab_Chemotherapy** | 50 | 21 | 10 | 6 | 4 | 3 | 2 | 1 | 1 | 1 | 0 | 0 | 0 | 0 | 0 | 0 | 0 | 0 | 0 | 0 | 0 | 0 | 0 | 0 | 0 | 0 | 0 |
| **Atezolizumab_Chemotherapy** | 0 | 0 | 0 | 0 | 0 | 0 | 1 | 1 | 2 | 3 | 4 | 6 | 9 | 11 | 13 | 14 | 13 | 9 | 6 | 4 | 2 | 1 | 0 | 0 | 0 | 0 | 0 |
| **Axitinib_Chemotherapy** | 4 | 5 | 5 | 5 | 5 | 5 | 5 | 5 | 5 | 5 | 5 | 5 | 5 | 5 | 5 | 5 | 4 | 4 | 3 | 3 | 2 | 2 | 1 | 1 | 1 | 1 | 0 |
| **Bevacizumab_Chemotherapy** | 0 | 0 | 0 | 2 | 4 | 8 | 13 | 16 | 16 | 15 | 11 | 7 | 4 | 2 | 1 | 0 | 0 | 0 | 0 | 0 | 0 | 0 | 0 | 0 | 0 | 0 | 0 |
| **Camrelizumab_Chemotherapy** | 3 | 7 | 10 | 12 | 13 | 12 | 10 | 8 | 7 | 5 | 4 | 3 | 2 | 1 | 1 | 1 | 0 | 0 | 0 | 0 | 0 | 0 | 0 | 0 | 0 | 0 | 0 |
| **Cediranib_Chemotherapy** | 2 | 5 | 7 | 9 | 10 | 10 | 9 | 9 | 8 | 7 | 6 | 5 | 4 | 3 | 2 | 1 | 1 | 1 | 0 | 0 | 0 | 0 | 0 | 0 | 0 | 0 | 0 |
| **Cemiplimab** | 1 | 3 | 4 | 5 | 6 | 7 | 7 | 7 | 7 | 7 | 7 | 7 | 6 | 5 | 5 | 4 | 3 | 2 | 2 | 1 | 1 | 0 | 0 | 0 | 0 | 0 | 0 |
| **Chemotherapy** | 0 | 0 | 0 | 0 | 0 | 0 | 0 | 0 | 0 | 0 | 0 | 0 | 0 | 0 | 0 | 0 | 0 | 0 | 2 | 7 | 17 | 26 | 25 | 16 | 6 | 1 | 0 |
| **Durvalumab** | 0 | 0 | 0 | 0 | 0 | 0 | 0 | 0 | 0 | 0 | 0 | 1 | 1 | 1 | 1 | 2 | 3 | 4 | 5 | 6 | 7 | 7 | 9 | 11 | 14 | 15 | 11 |
| **Durvalumab_Tremelimumab** | 0 | 0 | 0 | 0 | 0 | 0 | 0 | 0 | 0 | 0 | 0 | 0 | 1 | 1 | 1 | 1 | 2 | 3 | 4 | 5 | 6 | 7 | 8 | 11 | 15 | 18 | 16 |
| **Endostar_Chemotherapy** | 0 | 0 | 0 | 0 | 1 | 1 | 1 | 2 | 3 | 5 | 6 | 8 | 10 | 11 | 12 | 11 | 10 | 7 | 5 | 3 | 2 | 1 | 0 | 0 | 0 | 0 | 0 |
| **Ipilimumab_Chemotherapy** | 0 | 0 | 0 | 0 | 0 | 0 | 0 | 0 | 0 | 0 | 0 | 1 | 1 | 1 | 2 | 3 | 4 | 6 | 8 | 11 | 11 | 11 | 11 | 11 | 9 | 7 | 3 |
| **Motesanib_Chemotherapy** | 0 | 0 | 0 | 0 | 0 | 1 | 1 | 2 | 3 | 4 | 6 | 8 | 11 | 12 | 13 | 12 | 10 | 7 | 5 | 3 | 1 | 1 | 0 | 0 | 0 | 0 | 0 |
| **Nivolumab** | 0 | 0 | 0 | 0 | 0 | 0 | 0 | 0 | 0 | 0 | 0 | 0 | 0 | 0 | 0 | 0 | 1 | 1 | 2 | 2 | 3 | 4 | 5 | 9 | 13 | 20 | 39 |
| **Nivolumab_Bevacizumab_Chemotherapy** | 23 | 24 | 14 | 10 | 7 | 5 | 4 | 3 | 2 | 2 | 1 | 1 | 1 | 1 | 0 | 0 | 0 | 0 | 0 | 0 | 0 | 0 | 0 | 0 | 0 | 0 | 0 |
| **Nivolumab_Ipilimumab** | 2 | 4 | 6 | 8 | 8 | 9 | 9 | 8 | 8 | 7 | 7 | 6 | 5 | 4 | 3 | 2 | 2 | 1 | 1 | 1 | 0 | 0 | 0 | 0 | 0 | 0 | 0 |
| **Nivolumab_Ipilimumab_Chemotherapy** | 0 | 1 | 2 | 2 | 3 | 4 | 4 | 5 | 5 | 6 | 7 | 8 | 8 | 8 | 7 | 7 | 6 | 5 | 4 | 3 | 2 | 1 | 1 | 1 | 0 | 0 | 0 |
| **Pembrolizumab** | 0 | 0 | 0 | 0 | 0 | 0 | 0 | 0 | 1 | 1 | 1 | 2 | 3 | 4 | 6 | 8 | 11 | 15 | 15 | 12 | 8 | 5 | 4 | 2 | 1 | 1 | 0 |
| **Pembrolizumab_Chemotherapy** | 8 | 19 | 23 | 18 | 12 | 8 | 5 | 3 | 2 | 1 | 1 | 0 | 0 | 0 | 0 | 0 | 0 | 0 | 0 | 0 | 0 | 0 | 0 | 0 | 0 | 0 | 0 |
| **Pembrolizumab_Ipilimumab** | 0 | 0 | 0 | 1 | 1 | 1 | 1 | 1 | 2 | 2 | 2 | 3 | 3 | 4 | 5 | 5 | 6 | 8 | 9 | 8 | 7 | 6 | 6 | 6 | 6 | 5 | 4 |
| **Ramucirumab_Chemotherapy** | 1 | 2 | 3 | 4 | 5 | 5 | 6 | 6 | 6 | 7 | 7 | 7 | 7 | 7 | 6 | 5 | 5 | 4 | 3 | 2 | 1 | 1 | 1 | 0 | 0 | 0 | 0 |
| **Sintilimab_Chemotherapy** | 0 | 0 | 1 | 2 | 2 | 4 | 5 | 5 | 7 | 8 | 9 | 9 | 10 | 9 | 8 | 7 | 5 | 4 | 3 | 2 | 1 | 0 | 0 | 0 | 0 | 0 | 0 |
| **Sorafenib_Chemotherapy** | 0 | 0 | 0 | 0 | 0 | 0 | 0 | 0 | 0 | 0 | 0 | 1 | 1 | 2 | 2 | 4 | 6 | 10 | 12 | 14 | 13 | 11 | 9 | 7 | 5 | 3 | 1 |
| **Sugemalimab_Chemotherapy** | 3 | 5 | 7 | 8 | 8 | 8 | 8 | 7 | 7 | 7 | 6 | 5 | 5 | 4 | 3 | 3 | 2 | 2 | 1 | 1 | 1 | 0 | 0 | 0 | 0 | 0 | 0 |
| **Thalidomide_Chemotherapy** | 0 | 0 | 0 | 0 | 0 | 0 | 0 | 0 | 0 | 0 | 0 | 0 | 1 | 1 | 1 | 2 | 2 | 4 | 5 | 7 | 8 | 8 | 10 | 12 | 14 | 14 | 11 |
| **Tislelizumab_Chemotherapy** | 2 | 4 | 7 | 9 | 10 | 10 | 10 | 9 | 8 | 7 | 6 | 5 | 4 | 3 | 2 | 1 | 1 | 1 | 0 | 0 | 0 | 0 | 0 | 0 | 0 | 0 | 0 |

|  | **V1** | **V2** | **V3** | **V4** | **V5** | **V6** | **V7** | **V8** | **V9** | **V10** | **V11** | **V12** | **V13** | **V14** | **V15** | **V16** | **V17** | **V18** | **V19** | **V20** | **V21** | **V22** | **V23** | **V24** | **V25** | **V26** |
| --- | --- | --- | --- | --- | --- | --- | --- | --- | --- | --- | --- | --- | --- | --- | --- | --- | --- | --- | --- | --- | --- | --- | --- | --- | --- | --- |
| **Progression free survival** | | | | | | | | | | | | | | | | | | | | | | | | | | |
| **Atezolizumab** | 0 | 1 | 1 | 1 | 2 | 2 | 3 | 4 | 5 | 5 | 6 | 6 | 6 | 6 | 6 | 6 | 6 | 6 | 5 | 5 | 4 | 4 | 3 | 3 | 3 | 1 |
| **Atezolizumab_Bevacizumab_Chemotherapy** | 21 | 19 | 13 | 10 | 8 | 7 | 5 | 4 | 3 | 2 | 2 | 1 | 1 | 1 | 1 | 0 | 0 | 0 | 0 | 0 | 0 | 0 | 0 | 0 | 0 | 0 |
| **Atezolizumab_Chemotherapy** | 0 | 0 | 1 | 2 | 4 | 6 | 9 | 11 | 13 | 13 | 11 | 9 | 7 | 5 | 4 | 3 | 2 | 1 | 1 | 0 | 0 | 0 | 0 | 0 | 0 | 0 |
| **Axitinib_Chemotherapy** | 1 | 1 | 1 | 1 | 1 | 2 | 2 | 3 | 3 | 3 | 3 | 4 | 4 | 4 | 4 | 4 | 5 | 5 | 5 | 5 | 5 | 5 | 6 | 7 | 8 | 9 |
| **Bevacizumab_Chemotherapy** | 0 | 0 | 0 | 0 | 0 | 0 | 1 | 2 | 4 | 7 | 10 | 12 | 13 | 13 | 11 | 9 | 7 | 5 | 3 | 2 | 1 | 0 | 0 | 0 | 0 | 0 |
| **Camrelizumab_Chemotherapy** | 13 | 17 | 16 | 14 | 11 | 9 | 7 | 5 | 3 | 2 | 1 | 1 | 1 | 0 | 0 | 0 | 0 | 0 | 0 | 0 | 0 | 0 | 0 | 0 | 0 | 0 |
| **Cediranib_Chemotherapy** | 0 | 0 | 0 | 0 | 0 | 1 | 1 | 2 | 2 | 3 | 4 | 5 | 5 | 6 | 7 | 8 | 8 | 8 | 8 | 7 | 6 | 5 | 5 | 4 | 3 | 1 |
| **Cemiplimab** | 3 | 5 | 6 | 7 | 7 | 8 | 9 | 8 | 8 | 7 | 6 | 5 | 4 | 3 | 3 | 2 | 2 | 2 | 1 | 1 | 1 | 1 | 1 | 0 | 0 | 0 |
| **Chemotherapy** | 0 | 0 | 0 | 0 | 0 | 0 | 0 | 0 | 0 | 0 | 0 | 0 | 0 | 0 | 0 | 0 | 1 | 3 | 7 | 14 | 22 | 24 | 18 | 8 | 2 | 0 |
| **Durvalumab** | 0 | 0 | 0 | 1 | 1 | 1 | 1 | 2 | 2 | 3 | 3 | 4 | 4 | 4 | 5 | 5 | 6 | 6 | 6 | 6 | 6 | 6 | 7 | 8 | 7 | 4 |
| **Durvalumab_Tremelimumab** | 0 | 0 | 0 | 0 | 0 | 0 | 0 | 0 | 1 | 1 | 1 | 1 | 1 | 2 | 2 | 3 | 3 | 4 | 4 | 5 | 5 | 6 | 9 | 13 | 17 | 20 |
| **Ipilimumab_Chemotherapy** | 0 | 0 | 0 | 0 | 0 | 0 | 0 | 0 | 1 | 1 | 1 | 2 | 3 | 4 | 4 | 6 | 7 | 8 | 9 | 9 | 9 | 9 | 9 | 9 | 7 | 3 |
| **Motesanib_Chemotherapy** | 0 | 0 | 0 | 0 | 0 | 0 | 1 | 1 | 2 | 3 | 4 | 6 | 7 | 8 | 10 | 10 | 10 | 10 | 8 | 6 | 5 | 3 | 2 | 2 | 1 | 0 |
| **Nivolumab** | 0 | 0 | 0 | 0 | 0 | 0 | 0 | 0 | 0 | 0 | 0 | 1 | 1 | 1 | 1 | 2 | 2 | 3 | 3 | 4 | 4 | 6 | 8 | 13 | 19 | 31 |
| **Nivolumab_Bevacizumab_Chemotheray** | 37 | 18 | 11 | 8 | 6 | 5 | 4 | 3 | 2 | 2 | 1 | 1 | 1 | 1 | 0 | 0 | 0 | 0 | 0 | 0 | 0 | 0 | 0 | 0 | 0 | 0 |
| **Nivolumab_Ipilimumab** | 2 | 3 | 4 | 5 | 6 | 7 | 8 | 8 | 8 | 7 | 7 | 6 | 5 | 4 | 4 | 3 | 3 | 2 | 2 | 1 | 1 | 1 | 1 | 1 | 0 | 0 |
| **Nivolumab_Ipilimumab_Chemotherapy** | 1 | 2 | 2 | 3 | 4 | 5 | 6 | 7 | 7 | 7 | 7 | 7 | 6 | 6 | 5 | 5 | 4 | 4 | 3 | 2 | 2 | 2 | 1 | 1 | 1 | 0 |
| **Pembrolizumab** | 0 | 0 | 0 | 0 | 1 | 1 | 2 | 2 | 3 | 5 | 6 | 7 | 7 | 8 | 9 | 9 | 8 | 8 | 7 | 5 | 4 | 3 | 2 | 2 | 1 | 0 |
| **Pembrolizumab_Chemotherapy** | 6 | 12 | 17 | 18 | 16 | 12 | 8 | 5 | 3 | 2 | 1 | 0 | 0 | 0 | 0 | 0 | 0 | 0 | 0 | 0 | 0 | 0 | 0 | 0 | 0 | 0 |
| **Pembrolizumab_Ipilimumab** | 1 | 1 | 1 | 1 | 2 | 2 | 2 | 3 | 3 | 4 | 4 | 4 | 4 | 5 | 5 | 5 | 5 | 5 | 5 | 5 | 5 | 5 | 5 | 6 | 6 | 5 |
| **Ramucirumab_Chemotherapy** | 0 | 0 | 0 | 1 | 1 | 1 | 2 | 3 | 3 | 4 | 5 | 6 | 6 | 7 | 7 | 7 | 7 | 7 | 6 | 6 | 5 | 4 | 4 | 4 | 3 | 1 |
| **Sintilimab_Chemotherapy** | 6 | 9 | 12 | 12 | 13 | 12 | 10 | 8 | 6 | 4 | 3 | 2 | 1 | 1 | 1 | 0 | 0 | 0 | 0 | 0 | 0 | 0 | 0 | 0 | 0 | 0 |
| **Sorafenib_Chemotherapy** | 0 | 0 | 0 | 0 | 0 | 0 | 0 | 1 | 1 | 1 | 2 | 3 | 3 | 4 | 6 | 7 | 8 | 9 | 10 | 9 | 9 | 8 | 7 | 6 | 4 | 2 |
| **Sugemalimab_Chemotherapy** | 8 | 9 | 9 | 9 | 9 | 8 | 8 | 7 | 6 | 5 | 4 | 3 | 3 | 2 | 2 | 2 | 1 | 1 | 1 | 1 | 1 | 0 | 0 | 0 | 0 | 0 |
| **Thalidomide_Chemotherapy** | 0 | 0 | 0 | 0 | 0 | 0 | 0 | 0 | 0 | 1 | 1 | 1 | 1 | 2 | 2 | 2 | 3 | 4 | 4 | 5 | 6 | 7 | 10 | 13 | 18 | 20 |
| **Tislelizumab_Chemotherapy** | 1 | 3 | 5 | 7 | 8 | 10 | 11 | 11 | 10 | 8 | 6 | 5 | 4 | 3 | 2 | 2 | 1 | 1 | 1 | 0 | 0 | 0 | 0 | 0 | 0 | 0 |

**Supplementary.Table.2** Comparisons of the fit of consistency and inconsistency models

| **Model** | **Progression-free survival** | **Overall survival** | **Objective response rate** | **Rate of grade3-4 toxicity assessment** |
| --- | --- | --- | --- | --- |
| **Consistency** | 90.59733 | 78.93336 | 201.55996 | 138.10159 |
| **Inconsistency** | 90.58719 | 79.24906 | 201.37488 | 138.33259 |

| **Model** | **Disease controlled rate** | **Adverse events---all grades** | **Adverse events---leading to discontinuation** | **Adverse events---leading to death** |
| --- | --- | --- | --- | --- |
| **Consistency** | 135.58961 | 83.76441 | 131.61738 | 106.24535 |
| **Inconsistency** | 135.78762 | 83.61683 | 131.46059 | 106.45542 |

| **Model** | **Anemia** | **Neutropenia** | **Thrombocytopenia** | **Fatigue** | **Diarrhea** | **Nausea/vomiting** | **Asthenia** |
| --- | --- | --- | --- | --- | --- | --- | --- |
| **Consistency** | 145.64747 | 140.09409 | 140.35567 | 96.78622 | 104.21441 | 132.32831 | 61.43538 |
| **Inconsistency** | 145.59709 | 139.66875 | 140.00149 | 96.15256 | 104.08458 | 132.08459 | 61.51352 |

| **PDL-1 expression status** | **Negative** | | **Intermediate** | | **High** | |
| --- | --- | --- | --- | --- | --- | --- |
| **Model** | **Progression free survival** | **Overall survival** | **Progression free survival** | **Overall survival** | **Progression free survival** | **Overall survival** |
| **Consistency** | 30.26613 | 19.35445 | 26.61541 | 17.284474 | 34.53735 | 22.26697 |
| **Inconsistency** | 30.17210 | 19.316987 | 26.68219 | 17.204266 | 34.68163 | 22.23892 |
